# Assessing the Reliability of Truncated Coupled Cluster Wave Function: Estimating the Distance from the Exact Solution

**DOI:** 10.1021/acs.jctc.5c00750

**Published:** 2025-09-12

**Authors:** Ádám Ganyecz, Zsolt Benedek, Klára Petrov, Gergely Barcza, András Olasz, Miklós A. Werner, Örs Legeza

**Affiliations:** † Strongly Correlated Systems “Lendület” Research Group, 162159 Wigner Research Centre for Physics, H-1525, Budapest, Hungary; ‡ Department of Physics of Complex Systems, 54616 Eötvös Loránd University, Egyetem tér 1-3, H-1053 Budapest, Hungary; § MTA−ELTE Lendület “Momentum” NewQubit Research Group, Pázmány Péter, Sétány 1/A, 1117, Budapest, Hungary; ∥ Department of Physical Chemistry and Materials Science, Faculty of Chemical Technology and Biotechnology, Budapest University of Technology and Economics, Müegyetem rkp. 3., H-1111, Budapest, Hungary; ⊥ Furukawa Electric Institute of Technology Ltd., Késmárk street 28/A, H-1158, Budapest, Hungary; # Institute for Advanced Study, Technical University of Munich, Lichtenbergstrasse 2a, 85748, Garching, Germany; ○ Parmenides Stiftung, Hindenburgstr. 15, 82343, Pöcking, Germany; 8 Dynaflex Ltd., H-1028 Budapest, Hungary

## Abstract

A new approach is proposed to assess the reliability of the truncated wave function methods by estimating the deviation from the full configuration interaction (FCI) wave function. While typical multireference diagnostics compare some derived property of the solution with the ideal picture of a single determinant, we try to answer a more practical question: how far is the solution from the exact one. Using the density matrix renormalization group (DMRG) method to provide an approximate FCI solution for the self-consistently determined relevant active space, we compare the low-level CI expansions and one-body reduced density matrixes to determine the distance of the two solutions (*d̃*_Φ_, *d̃*_γ_). We demonstrate the applicability of the approach for the CCSD method by benchmarking on the W4–17 data set, as well as on transition-metal-containing species. We also show that the presented moderate-cost, purely wave function-based metric is truly unique in the sense that it does not correlate with any popular multireference measures. We also explored the usage of CCSD natural orbitals (*d̃*_γ,NO_) and its effect on the active space size and the metric. The proposed diagnostic can also be applied to other wave function approximations, and it has the potential to provide a quality measure for post-Hartree–Fock procedures in general.

## Graphical abstract

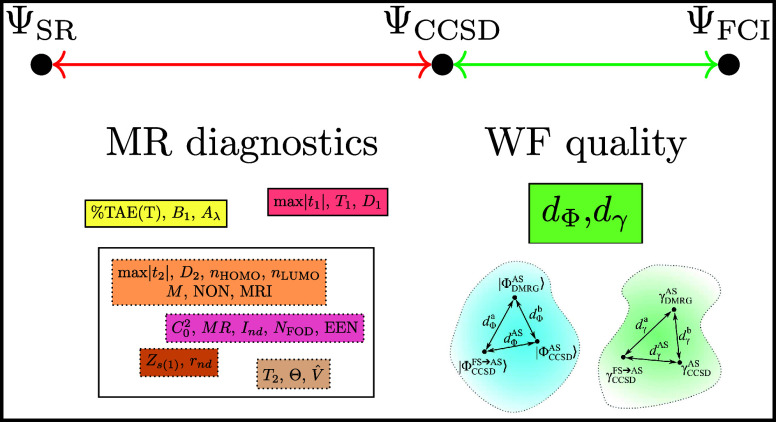

## 1 Introduction

In computational quantum chemistry, correlation effects are often distinguished into dynamic and static correlations according to their character.^1^ The latter one, also known as nondynamic correlation, can be further divided to Type A or left–right strong correlation and Type B or angular strong correlation.^2,3^ Type A correlation occurs when there is an absolute near-degeneracy, for example, bond stretching, while Type B refers to relative near-degeneracy when the gap is small compared to the orbital energies.

When dynamic correlation dominates, the routinely applied single reference (SR) quantum chemical methods, such as density functional theory (DFT) or the gold standard coupled cluster singles, doubles, and perturbative triples (CCSD­(T)), work well. However, they often do not provide accurate results for molecules with a significant nondynamic correlation.^4,5^ Therefore, measuring or at least estimating the degree of correlation is a crucial point in choosing the appropriate methodology. From the practical point of view in computational chemistry, it is also required that the cost of such a measurement should be comparable to that of a single reference calculation.^6^

To address this issue, numerous multireference (MR) diagnostics appeared in the literature^7–9^ that attempt to predict the validity of the single-reference approach by analyzing the SR results themselves. Only two of them are based on CI coefficients (*c*_*i*_): *C*_0_^2^ ^10,11^ is the weight of the leading determinant, usually from a CASSCF or CASCI calculation, while MR^12^ uses the second and fourth power of *c*_*i*_ coefficients to determine the deviation from the single determinant wave function.

Specifically, for CC methods, the most straightforward metrics were the maximum of *t*_1_ singles and *t*_2_ doubles amplitudes, which were available in the early versions of ACES quantum chemical program suite.^13,14^ The well-known *T*_1_ ^15^ and *D*_1_ ^16–18^ are based on the Euclidean and the Frobenius norm of *t*_1_ singles organized into the appropriate vector and matrix, respectively. Double excitation amplitudes can be used in a similar manner to obtain *T*_2_ ^19^ and *D*_2_ ^20^ for closed-shell systems. Most recently, the *S* diagnostic was proposed, which also uses Lagrange multipliers in addition to the cluster amplitudes.^21^

Another class of metrics is based on occupation numbers, usually of natural orbitals, which can be obtained by various methods. The simplest example uses the occupation number of HOMO and LUMO,^8,22,23^ which can be combined in one number, *M*.^22^ Coe and Paterson (based on Löwdin) defined Θ, which indicates how far the wave function is from the single-determinant solution of Hartree–Fock.^19,24^ Ramos-Cordoba et al. derived *I*_nd_ and *I*_d_ nondynamic and dynamic correlation indices based on the two-particle cumulant matrix.^25,26^ Kesharwani and co-workers defined an intensive quantity, *r*_nd_, as a ratio of nondynamic and total correlation indices, *I*_nd_/(*I*_nd_ + *I*_d_). Grimme and Hansen^27,28^ proposed a function called fractional occupation number weighted density (FOD) to visualize static or nondynamic correlation in the molecule. Its spatial integration produces a single number that can be used as an MR diagnostic metric. The fractional occupational numbers are determined with finite-temperature DFT in this approach. Bartlett et al.^29^ defined several new metrics for CC based on natural orbital occupation numbers, determined from 1-body RDM, but these definitions can also be used with any occupation number. The external electron number (EEN) shows how many electrons are in the virtual orbitals; its counterpart, the variance relative to the ideal single determinant case, *V̂*, is based on the occupation numbers of occupied orbitals and scaled with the number of orbitals. NON is the largest occupation number on unoccupied natural orbitals, which is also closely related to the LUMO occupation number. They also constructed a multireference index (MRI), transforming the occupation numbers to a single number ranging from −1 to 1, where MRI around −1 suggests MR character, while around +1 SR character is expected.

Following the work of Legeza and Sólyom on single orbital entropies^30^ and their sum providing total correlation^31^ Boguslawski and co-workers used these concepts to study multireference nature of strongly correlated molecules.^32^ Later, Stein and Reiher used total correlation to define *Z*_*s*(1)_ diagnostic which scales so that the maximal entanglement refers to 1 while no entanglement refers to the value 0.^33^

The next group of metrics can be called energy-based multireference diagnostics. These use some ratio of total atomization energies. %TAE­(T)^34,35^ measures the effect of perturbative triples on total atomization energy. *B*_1_ ^36^ uses the energy difference of BLYP^37,38^ and B1LYP^39^ and scales with the number of bonds. *A*_λ_ ^8^ takes the difference between the pure and its hybrid DFT version and is scaled with λ, which is a percent of the HF-exchange of the hybrid functional. Recently, Martin and his co-workers proposed %TAE_*X*_,^40^ which measures for a given HF density the difference of the DFT and HF exchange energies. The various multireference diagnostics used in this work are summarized in Table 1.

**Table 1:** List of MR Diagnostics Studied in This Work

name	type	definition^a^	this work^b^	ref
*C*_0_^2^	CI coefficient	square of coefficient of leading determinant	CCSD, DMRG	^10^,^11^
*MR*	CI coefficient	$\underset{i}{\sum }\left|c_{i}{|}^{2}-\right|c_{i}{|}^{4}$	CCSD, DMRG	^12^
max|*t*_1_|	CC amplitudes	maximum of *t*_1_ amplitudes	CCSD	^13^
max|*t*_2_|	CC amplitudes	maximum of *t*_2_ amplitudes	CCSD	^13^
*T*_1_	CC amplitudes	$\mathrm{||}t_{1}{\mathrm{||}}_{F}/\sqrt{n_{\mathrm{corr}}}$	CCSD	^15^
*T*_2_	CC amplitudes	$\mathrm{||}t_{2}{\mathrm{||}}_{F}/\sqrt{n_{\mathrm{corr}}}$	CCSD	^19^
*D*_1_	CC amplitudes	||**T**_1_||_2_	CCSD	^16–18^
*D*_2_	CC amplitudes	max(||**T**_2_^*o*^||_2_, ||**T**_2_^*v*^||_2_), where $T_{2}^{o}\in R^{{\mathrm{ov}}^{2}\times o}$ and $T_{2}^{v}\in R^{o^{2}v\times v}$	CCSD	^20^
*n*_HOMO_	occupation numbers	occupation number of HOMO	CCSD, DMRG, FT-DFT	^8^,^22^,^23^
*n*_LUMO_	occupation numbers	occupation number of LUMO	CCSD, DMRG, FT-DFT	^8^,^22^,^23^
*M*	occupation numbers	$\frac{1}{2}\left(2-n_{\mathrm{HOMO}}+n_{\mathrm{LUMO}}+\overset{N_{\mathrm{SOMO}}}{\underset{j}{\sum }}|n_{j}-1|\right)$	CCSD, DMRG, FT-DFT	^22^
*I*_nd_	occupation numbers	$\frac{1}{2}\underset{\sigma ,i}{\sum }n_{i}^{\sigma }\left(1-n_{i}^{\sigma }\right)$	CCSD, DMRG, FT-DFT	^25^,^26^
*r*_nd_	occupation numbers	$\frac{\frac{1}{2}\underset{\sigma ,i}{\sum }n_{i}^{\sigma }\left(1-n_{i}^{\sigma }\right)}{\frac{1}{4}\underset{\sigma ,i}{\sum }{\left[n_{i}^{\sigma }\left(1-n_{i}^{\sigma }\right)\right]}^{1/2}}$	CCSD, DMRG, FT-DFT	^41^
Θ	occupation numbers	$1-\frac{1}{n}\underset{i,\sigma }{\sum }n_{i}^{\sigma 2}$	CCSD, DMRG, FT-DFT	^19^,^24^
*N*_FOD_	occupation numbers	$\overset{N_{\mathrm{occ}}}{\underset{i}{\sum }}1-n_{i}^{\sigma }+\overset{N_{\mathrm{virt}}}{\underset{j}{\sum }}n_{j}^{\sigma }$	CCSD, DMRG, FT-DFT	^27^,^28^
EEN	occupation numbers	$\overset{N_{\mathrm{virt}}}{\underset{i}{\sum }}n_{i}^{\sigma }$	CCSD, DMRG, FT-DFT	^29^
V̂	occupation numbers	$\frac{1}{n}\left(\overset{N_{\mathrm{occ}}}{\underset{i}{\sum }}n_{i}^{\sigma }-\overset{N_{\mathrm{occ}}}{\underset{i}{\sum }}n_{i}^{\sigma 2}\right)$	CCSD, DMRG, FT-DFT	^29^
MRI	occupation numbers	MRI=−tanh(4.016+log(I))	CCSD, DMRG, FT-DFT	^29^
		$I=\underset{i}{\sum }\exp\left(-1500\times {\left(0.5-n_{i}\right)}^{6}\right)$		
NON	occupation numbers	largest occupation number on unoccupied natural orbitals	CCSD, DMRG, FT-DFT	^29^
*Z*_*s*(1)_	orbital entropy	$\frac{1}{N\ln4}\overset{N}{\underset{i}{\sum }}s_{i}\left(1\right)$	CCSD, DMRG	^33^
%TAE(T)	energy	$100\times \frac{\mathrm{TAE}\left[\mathrm{CCSD}\left(T\right)\right]-\mathrm{TAE}\left[\mathrm{CCSD}\right]}{\mathrm{TAE}\left[\mathrm{CCSD}\right]}$	CCSD(T)	^34^,^35^
*B*_1_	energy	$\frac{\mathrm{TAE}\left[\mathrm{BLYP}\right]-\mathrm{TAE}\left[B1\mathrm{LYP}\right]}{n_{\mathrm{bonds}}}$	BLYP, B1LYP	^36^
*A*_λ_	energy	$\frac{100}{\lambda }\frac{\mathrm{TAE}\left[\mathrm{XC}\right]-\mathrm{TAE}\left[X_{\lambda }C\right]}{\mathrm{TAE}\left[\mathrm{XC}\right]}$	PBE, PBE0	^8^

a *N* (*N*_occ_, *N*_virt_, *N*_SOMO_): number of orbitals (occupied, virtual, single occupied), *n*_(corr)_: number of (correlated) electrons, *n*_*i*_ (*n*_*i*_^σ^ *n*_HOMO_, *n*_LUMO_): occupational number (spinorbitals, HOMO, LUMO) *c*_*i*_: CI coefficient, *t*_1_, *t*_2_: single/double excitation CC amplitudes, TAE: total atomization energy.

b Calculations used in this work to determine the metric.

Even though numerous diagnostics exist, their accuracy in predicting which system should not be handled with single reference methods is often questionable. Usually, they do not even correlate with each other.^6,8,40^ This leads to arbitrary rules where multiple diagnostics are used, such as species with 3d transition metals are considered multireference if *T*_1_ > 0.05, *D*_1_ > 0.15, and %TAE­[(T)] > 10%.^7^

All of the aforementioned metrics try to determine the degree of multireference character by taking the ideal single reference wave function and measuring the deviation from it. However, the real question is how far the solution of a given method is from the exact full CI wave function.

In this work, we propose a new family of metrics, *d̃*, which represents the quality of the wave function by measuring its deviation from the reference obtained by high-level theory. *d̃*_Φ_ uses CI-coefficients up to double excitations, while *d̃*_γ_ uses the 1-body reduced density matrix, and *d̃*_γ,NO_ uses also the natural orbital transformation to reduce the size of the active space used. First, we present the formulation of the new metrics. Then, we show the validity of the introduced approximations. After that, we compare the performance of *d̃*-s to other popular multireference diagnostics on the W4–17 data set, and also, a grouping of existing metrics is presented. The performance is also demonstrated on transition metal species, which are particularly difficult cases for standard single reference methods.

## 2 Theory

In this section, we present how the new metrics are formulated. First, we discuss the reference used and then which property will be used as a basis of comparison. After that we discuss the orbital selection procedure and then present the definition of the final metric.

### 2.1 Reference

Testing the quality of approximate quantum chemical methods, such as coupled cluster theory, against the exact reference provided by FCI is possible up to only 20 orbitals due to its computational demand.^42^ To overcome this limitation, in this paper, we applied the density matrix renormalization group (DMRG) approach^43^ as a robust quasi-FCI solver with polynomial scaling. The reference for all the investigated quantities was derived from the high-precision DMRG wave function whose accuracy was controlled by the truncation error.^44^ For a practical review of the DMRG method, see refs 45–53 .

### 2.2 Basis of Comparison

There are numerous ways to compare wave functions. The easiest way would be to use energy or some other property. However, in this case, we would lose most of the information that is contained in the wave function. If the wave function is expressed in MO-based Slater-determinants, then one can use the CI-coefficients (*c*_*i*_) as a basis of comparison. Due to the exponential formulation of CC wave functions, studied in our work in detail, contributions for all higher excited determinants are present even for CC truncated to single and double (SD) excitations. The expression of the full *c*_*i*_ is, however, numerically infeasible for the systems of our interest, as it would be as large as the FCI wave function, therefore it should also be truncated. Additionally, due to the internal normalization of the CC wave function, we can not determine the error caused by this truncation, unless we evaluate all coefficients. Consequently, the comparison based on the CI coefficients will be restricted up to double excitations by projecting the wave functions to this subspace and then normalizing them,

1 $$\left|\Phi \right\rangle =\frac{\mathrm{P̂}\left|\Psi \right\rangle }{||\mathrm{P̂}\left|\Psi \right\rangle {||}_{2}}$$

where |Ψ⟩ is the wave function expressed as the linear combination of all possible configurations. In the following, |Φ⟩ will denote the wave function projected on the basis of the reference determinant and the corresponding single and double excited configurations and then normalized, *P̂* projects to the subspace of single and double excitations, and || ||_2_ stands for the usual norm of the wave function.

Reduced density matrices provide an alternative way to represent the quantum state. The one-particle reduced density matrix (1-RDM, γ) contains partial, compressed information about the wave function, and will also be used as an alternative basis of comparison. As CC is not a variational method, different formulations exist for the density matrices. In this work we used unrelaxed RDMs, i.e., response RDMs without orbital relaxaton effects.

As a distance metric, we will use the Euclidean norm of the differences of the normalized vector made of *c* coefficients (Ψ), or their projection *c* ^SD^ up to doubles excitation (Φ), or the Hilbert–Schmidt norm of the differences of 1-body RDM matrices (γ). The distance from the reference wave function or RDM is denoted by *d*, while the basis of comparison is represented in the lower index.

2a $$d_{\Psi }=∥\Psi_{\mathrm{CCSD}}-\Psi_{\mathrm{DMRG}}∥$$

2b $$d_{\Phi }=∥\Phi_{\mathrm{CCSD}}-\Phi_{\mathrm{DMRG}}∥$$

2c $$d_{\gamma }=∥\gamma_{\mathrm{CCSD}}-\gamma_{\mathrm{DMRG}}∥$$

We note that *d*_Ψ_ is only available for small systems, but *d*_Φ_ and *d*_γ_ can be evaluated efficiently for larger systems, too. In the following, if there is no subscript following *d*, we refer to the distance metric in general.

### 2.3 Active Space Selection

Although DMRG can handle active spaces close to a hundred orbitals,^49,52,54–58^ the accessible number of orbitals is still lower than in CCSD, therefore the orbital space should be truncated.

A universally applicable black-box active space selection strategy is challenging to design. However, an automatized selection protocol based on single orbital entropies^30^ introduced via the dynamically extended active space (DEAS) procedure, also utilized in a more general framework,^33,59^ can offer a reasonable solution. In this procedure, an initial, low bond dimension DMRG is performed on the full valence space, and the active space is selected based on single-orbital entropies with an empirical preset cutoff relative to the highest observed entropy value.

In this study, we develop an alternative entropy-based selection. As a first step of the entire workflow, we perform a standard CCSD (or any other post-HF) calculation, which we later want to analyze and from which the Φ or γ can be easily extracted (light blue part of Figure 3). Recall that the single-orbital entropy^30^ for orbital *i* is defined as

3 $$s_{i}\left(1\right)=-\underset{\alpha }{\sum }p_{i}^{\alpha }\ln p_{i}^{\alpha }$$

where *p*_*i*_^α^ denotes the probability that orbital *i* is found in occupation state α ∈ {0, ↓, ↑, ↓↑} in the many-body wave function. The *p*_*i*_^α^ weights can be determined by summing the square of the CI coefficients of the corresponding determinants. We note that in practical CC calculations, we estimate the probabilities by restricting the CI expansion of the wave function up to double excitations. Alternatively, exact entropies could also be constructed from relevant entries of the 1- and 2-body reduced density matrices;^60–62^ however, this requires solving the Λ equations besides the CC amplitude equations, which both have similar costs, while for the calculation of the energy the solution of the CC amplitude equations are sufficient.

By sorting the *s*_*i*_(1) values in descending order, the orbitals can be selected according to their chemical relevance, where orbitals with the highest entropies will form the active space.

However, the question remains how to choose the cutoff of *s*_*i*_(1) between the active and inactive orbitals. Here, instead of predefining a fixed *s*_*i*_(1) value or proportion, such as for example 10% in the AutoCAS program,^33^ we apply a different approach. Based on the sorted *s*_*i*_(1) values, we identify various subsets of orbitals by their importance, where active orbitals are distinguished from inactive ones by the rate of the change in the entropy profile. More precisely, orbitals labeled {1, ..., *i*} in the descending entropy order define an active space in case of *s*_*i*–2_(1) – *s*_*i*–1_(1) < *s*_*i*–1_(1) – *s*_*i*_(1) (i.e., the entropy difference between two adjacent orbitals is larger than previously).

In some way, we try to separate the static and dynamic correlation. In that sense, we are interested in how well the static correlation is described by the selected active space and not in the dynamic correlation mostly captured by the inactive orbitals. Once the possible active spaces are determined, starting from the smallest ones, we perform CCSD calculation for the selected active space, which results are marked by superscript AS. The obtained CCSD wave function (Φ_CCSD_^AS^) or RDM (γ_CCSD_^AS^) is compared to the corresponding projection of the full space CCSD wave function (Φ_CCSD_^FS→AS^ or γ_CCSD_^FS→AS^). The corresponding distances are

4a $$d_{\Phi }^{\mathrm{AS}}=∥\Phi_{\mathrm{CCSD}}^{\mathrm{AS}}-\Phi_{\mathrm{CCSD}}^{\mathrm{FS}\to \mathrm{AS}}∥$$

4b $$d_{\gamma }^{\mathrm{AS}}=∥\gamma_{\mathrm{CCSD}}^{\mathrm{AS}}-\gamma_{\mathrm{CCSD}}^{\mathrm{FS}\to \mathrm{AS}}∥$$

Note that the full space Φ_CCSD_ and γ_CCSD_ are projected to the selected subspace, where only those configurations are kept, in which excitations are allowed for orbitals in the active space, hence, the “FS → AS” in the upper index. The wave function and RDM are normalized to give 1 and the correct electron number, respectively.

By comparing wave functions of the full space CCSD and the one of the CCSD on active space, *d* ^AS^ measures the error of active space selection. By definition, *d* ^AS^ equals zero if all orbitals are active. Hence, the active space can be considered as converged once *d* ^AS^ falls below a predefined threshold. If *d* ^AS^ is larger than the desired threshold, we continue with including the next batch of orbitals in the active space proposed by the entropy profile until the defined criteria are satisfied. This selection approach ensures that we select a subspace that correctly represents the whole space. For small active spaces, it might happen that first *d* ^AS^ increases (see later), therefore we apply a conservative approach, and *d* ^AS^ have to be not only below a threshold, but have to be decreasing (be smaller than the previous iteration). The whole active space selection is summarized by the light pink part of Figure 3.

The relation of the various wave functions is summarized in Figure 1, using BN at CCSD/cc-pVDZ level of theory as an example. First, we have Ψ_CCSD_^FS^ the full space CCSD wave function (accessible only for small systems). Though the number of cluster amplitudes are limited, due to the exponential ansatz, the CI coefficients are not. The evaluation of all coefficients is not practical, and it is limited up to double excitations; higher excitations, represented in blue shade, are ignored, and the error introduced with this truncation is unknown. Φ_CCSD_^FS^ is the wave function with only single and double excitations, while Φ_CCSD_^FS→AS^ contains only the excitations that are in the active space. The error of this truncation is the yellow shaded area in the figure. Finally, we compare this wave function to Φ_CCSD_^AS^, to get *d*_Φ_^AS^. We note that in the case of γ, there is only active space truncation and no exclusion of higher excitations.

**Figure 1:**
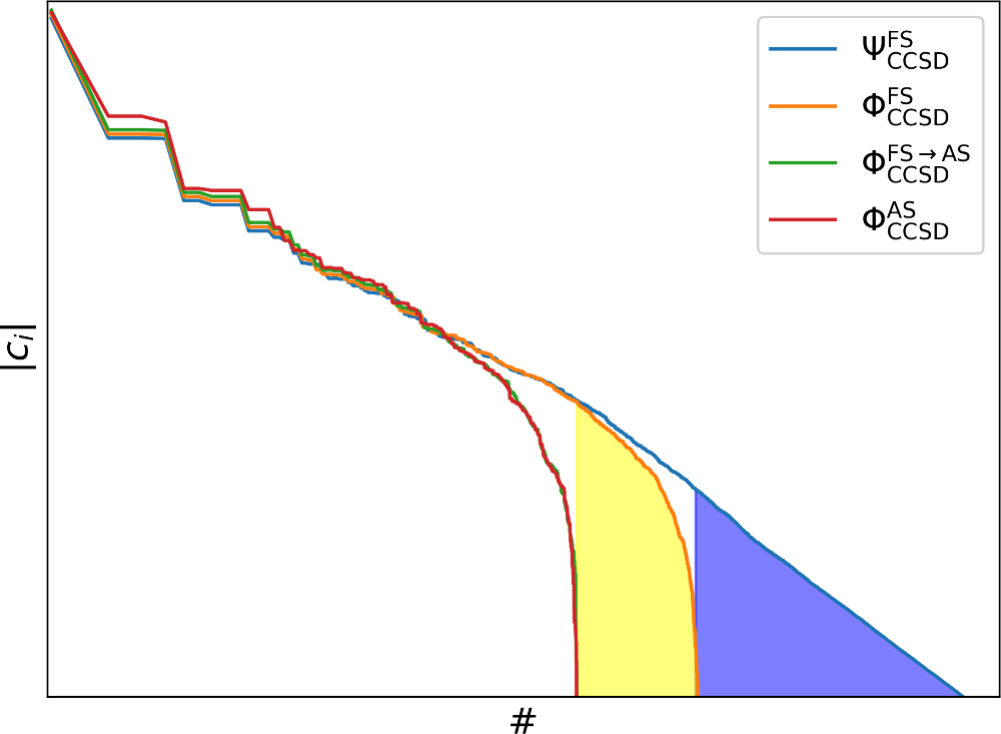
Schematic representation of the different normalized CCSD wave functions by plotting the corresponding CI coefficients for BN at the CCSD/cc-pVDZ level of theory and AS represents 14 orbitals. The blue shaded part represents the part of the wave function that is discarded by restricting up to double excitations. The yellow shaded part shows the determinants that are discarded due to the active space truncation.

### 2.4 Forming the Final Metric

Following the active space selection, a DMRG calculation is performed. This produces a third wave function, besides the existing active space and full space CCSD wave functions, which wave function we represent by its CI coefficients, *c*, or 1-RDM, γ. To ensure comparability, both three wave functions are vectors of the same active space and are normalized to one, and the electron number in 1-RDM’s is also properly set.

Between the three wave functions, three distances can be defined, and one of them, *d* ^AS^, is already defined in eq 4. It is easy to see that as the size of the active space approaches full space the wave function of the two CCSD calculation become more and more similar,

5a $$\underset{\mathrm{AS}\to \mathrm{FS}}{\lim}\Phi_{\mathrm{CCSD}}^{\mathrm{AS}}=\Phi_{\mathrm{CCSD}}^{\mathrm{FS}}$$

5b $$\underset{\mathrm{AS}\to \mathrm{FS}}{\lim}\gamma_{\mathrm{CCSD}}^{\mathrm{AS}}=\gamma_{\mathrm{CCSD}}^{\mathrm{FS}}$$

and, consequently,

6a $$\underset{\mathrm{AS}\to \mathrm{FS}}{\lim}d_{\Phi }^{\mathrm{AS}}=0$$

6b $$\underset{\mathrm{AS}\to \mathrm{FS}}{\lim}d_{\gamma }^{\mathrm{AS}}=0$$

It is important to note that the active space selection is based on the orbital entropies, meaning orbitals are included based on their importance in the wave function. The goal is to include all orbitals that are important to describe static correlations, while we aim to discard orbitals that are only contributing to the so-called dynamic correlation.

The other two are the distances from the DMRG solution,

7a $$d_{\Phi }^{a}=∥\Phi_{\mathrm{CCSD}}^{\mathrm{FS}\to \mathrm{AS}}-\Phi_{\mathrm{DMRG}}^{\mathrm{AS}}∥$$

7b $$d_{\gamma }^{a}=∥\gamma_{\mathrm{CCSD}}^{\mathrm{FS}\to \mathrm{AS}}-\gamma_{\mathrm{DMRG}}^{\mathrm{AS}}∥$$

and

8a $$d_{\Phi }^{b}=∥\Phi_{\mathrm{CCSD}}^{\mathrm{AS}}-\Phi_{\mathrm{DMRG}}^{\mathrm{AS}}∥$$

8b $$d_{\gamma }^{b}=∥\gamma_{\mathrm{CCSD}}^{\mathrm{AS}}-\gamma_{\mathrm{DMRG}}^{\mathrm{AS}}∥$$

Note again that in eq 7a the full space CCSD wave function has been projected to the active space and normalized to ensure comparability. At the full space limit, both of them are equal to *d*_Φ_ or *d*_γ_ value we are interested in

9a $$d_{\Phi }=∥\Phi_{\mathrm{CCSD}}^{\mathrm{FS}}-\Phi_{\mathrm{DMRG}}^{\mathrm{FS}}∥$$

9b $$d_{\gamma }=∥\gamma_{\mathrm{CCSD}}^{\mathrm{FS}}-\gamma_{\mathrm{DMRG}}^{\mathrm{FS}}∥$$

We can not choose from *d* ^a^ and *d* ^b^ which will be the better metric; therefore, both will be used. We note, however, that the triangle inequality holds for the 3 distances, and thus, the difference of *d* ^a^ and *d* ^b^ is bounded from above by *d* ^AS^,

10a $$\left|d_{\Phi }^{a}-d_{\Phi }^{b}\right|<d_{\Phi }^{\mathrm{AS}}$$

10b $$\left|d_{\gamma }^{a}-d_{\gamma }^{b}\right|<d_{\gamma }^{\mathrm{AS}}$$

The relation of various wave functions, RDMs and *d*-s introduces in this section are summarized in Figure 2. Here we remark, as will be discussed in the following sections, that in practice the average value of *d* ^*a*^ and *d* ^*b*^ will be used denoted by *d̃*.

**Figure 2:**
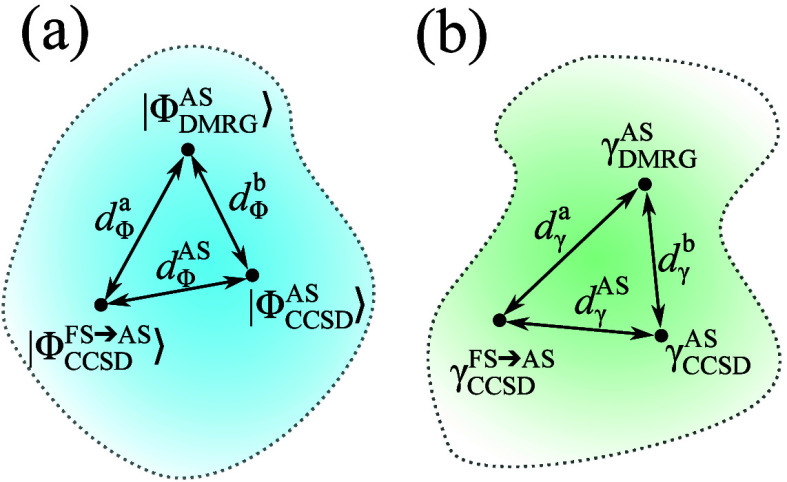
(a) Wave functions truncated to single and double excitations on the active space and the distances defined between them. (b) 1RDM-s on the active space and distances defined between them.

### 2.5 Possible Workflows

A general workflow is presented in Figure 3:

1. Perform CCSD calculation and obtain the basis of comparison (Φ_CCSD_^FS^ or γ_CCSD_^FS^).
2. Perform orbital transformation for better active space selection (optional).
3. Determine orbital entropies based on *c* or 1- and 2-RDM.
4. Select the most influential orbitals.
5. Perform CCSD on selected orbital space and obtain Φ_CCSD_^AS^ or γ_CCSD_^AS^.
6. Check if *d* ^AS^ is below threshold and smaller than the previous one (*d*_previous_^AS^), if yes, proceed, if not, repeat from step 4 by including more orbitals until true.
7. Perform orbital transformation for DMRG to aid better convergence (optional).
8. Perform DMRG and obtain Φ_DMRG_^AS^ or γ_DMRG_^AS^.
9. Detemine *d* ^a^ and *d* ^b^.

**Figure 3:**
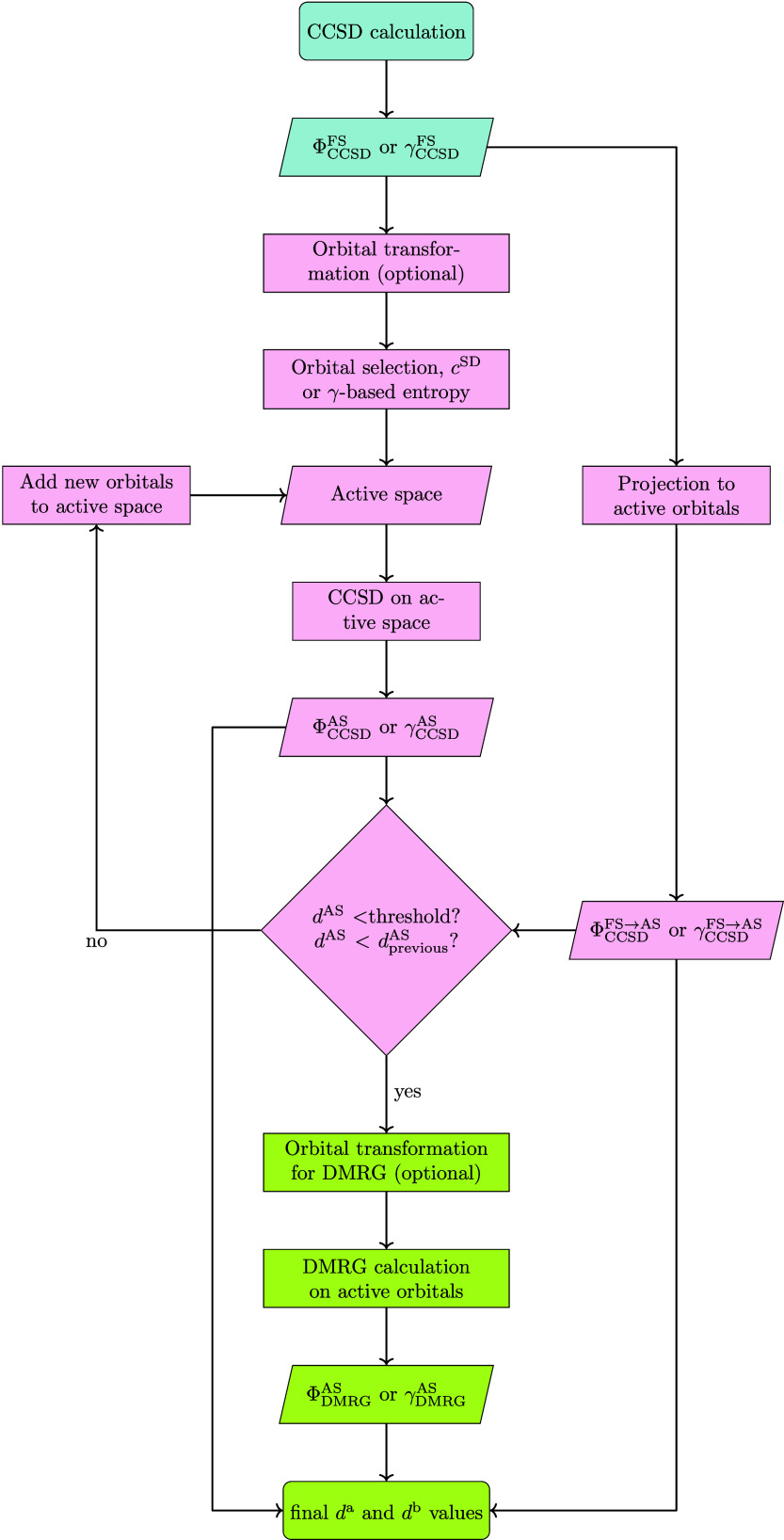
Flowchart of determination of the *d* diagnostic for a standard CCSD calculation. The different background coloring refers to the different steps, i.e., light blue: original CCSD calculation; light red: active space selection; light green: DMRG calculation and final *d* determination.

Besides the DMRG accuracy and the *d* ^AS^ threshold, we have to decide the basis of comparison also if we apply any orbital transformation. The threshold in *d* ^AS^ controls the accuracy of the final result, while the size of the active space strongly influences the cost of the DMRG calculation. Using some kind of orbital transformation before the orbital selection can compress correlations and can result in smaller active spaces. However, we have to ensure the invariance of CCSD solution under orbital transformations. The transformation before DMRG, like localization, can lead to better convergence. Based on the options, we define 3 workflows to apply in this work:

- Workflow A: Use Φ as a basis of comparison, no orbital transformation before selection, split-localization before DMRG. The corresponding result is denoted by *d*_Φ_.
- Workflow B: Use γ as a basis of comparison, no orbital transformation before selection, split-localization before DMRG. The corresponding result is denoted by *d*_γ_.
- Workflow C: Use γ as a basis of comparison, use “split-natural” orbitals for selection, no additional transformation before DMRG. The corresponding result is denoted by *d*_γ,NO_.

Workflow A is intended to apply when producing RDM-s would be too expensive. Workflow B is the same as A, but with γ instead of Φ, because it contains higher excitations from the wave function. Workflow C utilizes the fact that if the one-body RDM is at hand, then natural orbitals can be obtained. To keep the invariance of CCSD, the occupied and virtual orbitals should be transformed separately, also known as quasi-natural orbitals.^63^ We expect that the usage of natural orbitals compresses the correlation and, consequently, less orbitals are needed than in workflow B. In this case, we do not use localized orbitals, because DMRG works well with natural orbitals too.^64,65^

Importantly, even though the black-box computation of *d* requires two parameters (the *d* ^AS^ threshold and DMRG truncation error) as input, we emphasize that the choice of these parameters is rather flexible. That is, if doubts arise about the reliability of *d*, for example surprisingly high *d* value (over 0.5) or significantly higher energy with DMRG than CCSD for the same active space, more than 0.01 *E*_*h*_, a more accurate *d* can be computed at a larger active space and/or a lower truncation error.

Finally, we note that in the workflow both CCSD and DMRG can be substituted to any other method from which CI coefficients or the RDM can be extracted to estimate the distance between the wave functions of two methods.

## 3 Computational Details

In this section, we present computational details. Geometries for the W4–17 data set have been taken from the Supporting Information of ref 66, for the 3d-MLBE20 data set from ref 67. Furthermore, nine 3d and nine 4d transition metal species were studied from the work of Bradley et al.^68^ using geometries from refs 69–75 . These species will simply be termed 3d-TM9 and 4d-TM9.

As an additional challenge, we collected additional 3d and 4d species from the works of Jiang et al.^7^ and Wang et al.,^76^ which are claimed to be MR. Additionally, based on the work of Süß and co-workers^77^, species have also been added from the database of Aoto et al.,^78^ which have at least 2.5 kcal/mol correction to their bond dissociation energy with icMR-CCSD­(T) compared to CCSD­(T), along with the additional 12 systems studied by Süß et al. Geometries were optimized at B3LYP/cc-pVTZ level of theory, except for species from the database of Aoto et al.^78^ This collection of systems will be labeled as TM-MR. The full list of studied systems can be found in detail in Table S1.

CCSD^79^ and CCSD­(T)^80^ calculations were performed using cc-pVDZ^81–83^ basis set and frozen core approximation without density fitting with MRCC.^84^ A slight modification was made to obtain the CI coefficients from MRCC. Integrals for the subsequent DMRG calculation were also produced with MRCC. For open-shell species, the ROHF formalism was used.

The occupied and virtual orbitals were localized by Multiwfn^85^ applying the Pipek–Mezey localization scheme.^86^

All DMRG calculations were carried out with the Budapest-DMRG^87^ program package via the DBSS formalism,^44^ by setting the maximum truncation error, ϵ, to 10^–5^. The minimum value for the bond dimension was set to *D*_min_ = 256 and the maximum value was limited by *D*_max_ = 4096. For large active spaces DMRG orbital ordering was also optimized by a combination of the Fiedler-vector and genetic algorithm based protocols^88^ performing a series of low-cost DMRG calculations with fixed *D* = 32, 64, and 128 bond dimension values.

The various multireference diagnostics were already discussed in the Introduction; therefore, instead of a detailed description of each metric used in this work, we summarized them in Table 1.

Finite-temperature DFT calculations were performed with the default settings of the ORCA (version 5.0.3.^89^) program. All other DFT calculations (BLYP,^37,38^ B1LYP,^39^ PBE,^90^ PBE0 ^91^) were performed with MRCC.

The whole workflow is controlled by an in-house-developed Python code. Additional MR diagnostics were also evaluated by Python scripts processing DMRG and CCSD CI coefficients and DMRG and CCSD RDMs to obtain occupation numbers along with FT-DFT fractional occupation number, CCSD amplitudes, and necessary energies for energy-based diagnostics.

To better understand the practical relationship between the metrics, we determined the pairwise correlation coefficients between different data sets. The commonly used Pearson correlation (*r*), which lacks robustness and can reveal only linear correlations, is not the most suitable statistic to compare the metrics studied. Correspondingly, in our study Spearman (ρ) and Kendall rank correlations were used: (τ)

11 $$\rho =\frac{\mathrm{cov}(R\left[x\right]\left[R\left[y\right]\right)}{\rho_{R\left[x\right]}\rho_{R\left[y\right]}}$$

12 $$\tau =\frac{2}{n\left(n-1\right)}\underset{i<j}{\sum }\mathrm{sgn}\left(x_{i}-x_{j}\right)\mathrm{sgn}\left(y_{i}-y_{j}\right)$$

where *R*[*x*] and *R*[*y*] are the ranks of *x* and *y* variables, and *n* is the number data points. They are more reasonable statistics,^92^ which uses the ranking of the values and are more robust to outliers. Kendall correlation separates the various metrics even better. In short, we will refer to these correlation coefficients as *r*, ρ, and τ, respectively.

## 4 Results and Discussion

In order to keep the focus on the practical application of *d*, in this section only the main summary of the validation protocol is discussed, while details can be found in the SI.

The first approximation is the truncation of the Ψ wave function up to double excitations (Φ). The comparison of *d*_Ψ_ and *d*_Φ_ shows that *d*_Φ_ holds its descriptive power, although slightly lower than that of *d*_Ψ_.

The next approximation is the use of DMRG instead of FCI as an FCI solver. By varying the truncation error we found that at least a truncation error of 10^–5^ is needed for reasonable results.

The third approximation is the use of a subspace instead of the whole orbital set. In that sense, we checked the convergence of *d* ^AS^, *d* ^*a*^, and *d* ^*b*^ with respect to the active space size. In general, larger active space leads to lower *d* ^AS^, i.e., better quality. *d* ^*a*^ and *d* ^*b*^ converge smoothly to the full space *d* in most cases, but, it varies species by species which of them is more useful. However, we noticed that their average, labeled as *d̃*, has better convergence than *d* ^*a*^ and *d* ^*b*^ in every case; therefore, *d̃* will be used to estimate *d*. Another observation is that the NO-transformation can lead to smaller *d*-s by introducing a bias to the CCSD solution. These findings are valid for both the Φ- and γ-based protocols.

Based on these results, in the following, we will use two settings, a looser threshold of 0.1 and a tighter threshold of 0.05 for both *d*_Φ_^AS^ and *d*_γ_^AS^. The corresponding results will be noted as *d̃*_Φ_(0.1), *d̃*_Φ_(0.05), *d̃*_γ_(0.1), and *d̃*_γ_(0.05), where the number in the paranthesis is the used *d* ^AS^ threshold. An additional restriction is that the active space selection error should be lower than with the previous active space candidate to avoid a selection of active space before a local maximum.

### 4.1 Comparison of *d̃* and MR Diagnostics on the W4–17 Data Set

Before discussing the various *d̃* values, we take a look at the performance active space selection schemes. Using the looser 0.1 threshold obviously leads to smaller active spaces. As *d̃*_γ_ is usually higher than *d̃*_Φ_, to reach the same threshold on the same set of canonical orbitals, RDM-based orbital selection leads to larger active spaces, 43% and 37% of the whole orbital space on average with a threshold of 0.05 for *d*_γ_^AS^ and *d*_Φ_^AS^, respectively. However, as we have seen earlier, the usage of NO-transformation leads to less selected orbitals, 34% of the whole space on average if the threshold is 0.05. Note that these statistics are only valid for this data set; with larger basis sets we expect smaller ratios.

Instead of discussing the determined *d̃* values for the W4–17 data set alone, we will analyze them in relation to other existing MR diagnostics, which are listed in Table 1.

We think that both MR diagnostics and our *d̃* try to give guidance for the same problem; can we use a single reference method for a given system to obtain meaningful results? The difference is that MR diagnostics measure the MR character of the studied system, which is usually a metric of how different some property is from a reference, which usually is the ideal single reference wave function. In contrast, *d̃* takes a more practical approach and estimates the distance from the (approximate) FCI wave function. In line with their definition and philosophy, we expect a low correlation between MR diagnostics and *d̃*, but it is still worth comparing them since they deal with the same problem.

The quantum chemical method based on which we obtained the given diagnostic is presented in the corresponding superscript. For *C*_0_^2^ and *MR* we used the DMRG result instead of performing CASSCF. For metrics based on occupation numbers, CCSD- and DMRG-based RDMs were utilized to derive natural occupation numbers. Alternatively, fractional occupational numbers were also used from finite-temperature DFT. max|*t*_1_|, max|*t*_2_|, *T*_1_, *D*_1_, *T*_2_, and *D*_2_ are determined from CCSD/cc-pVDZ calculations, %TAE­(T) from CCSD­(T)/cc-pVDZ, *B*_1_ from BLYP/cc-pVDZ and B1LYP/cc-pVDZ, and *A*_25_ from PBE and PBE0 with cc-pVDZ, respectively.

The various MR diagnostic values for the MR subset are presented in Table 2. In cases where the definition allows multiple quantum chemical methods (e.g., metrics based on occupation numbers), we chose to show here the ones which are the closest to the definition in the original papers, while other versions can be found in the SI. For demonstrative purposes, we show herein all species where either *d̃* values are in the top 15 or one of the other diagnostics listed have a top 5 value among the species of the W4–17 data set. The threshold when a species is labeled as a multireference is not always defined and is sometimes debatable. Although we label the members of the “MR” subset, as given in the W4–17 paper,^66^ in Table 2 next to the molecular formula, we do not consider the W4–17-MR molecules as a specific group in our discussion. Instead, we rank the molecules of the W4–17 data set based on each investigated diagnostic value and use the ranking numbers as guidelines. This ranking is shown in the parentheses in Table 2 and is also visualized by the coloring.

**Table 2:**
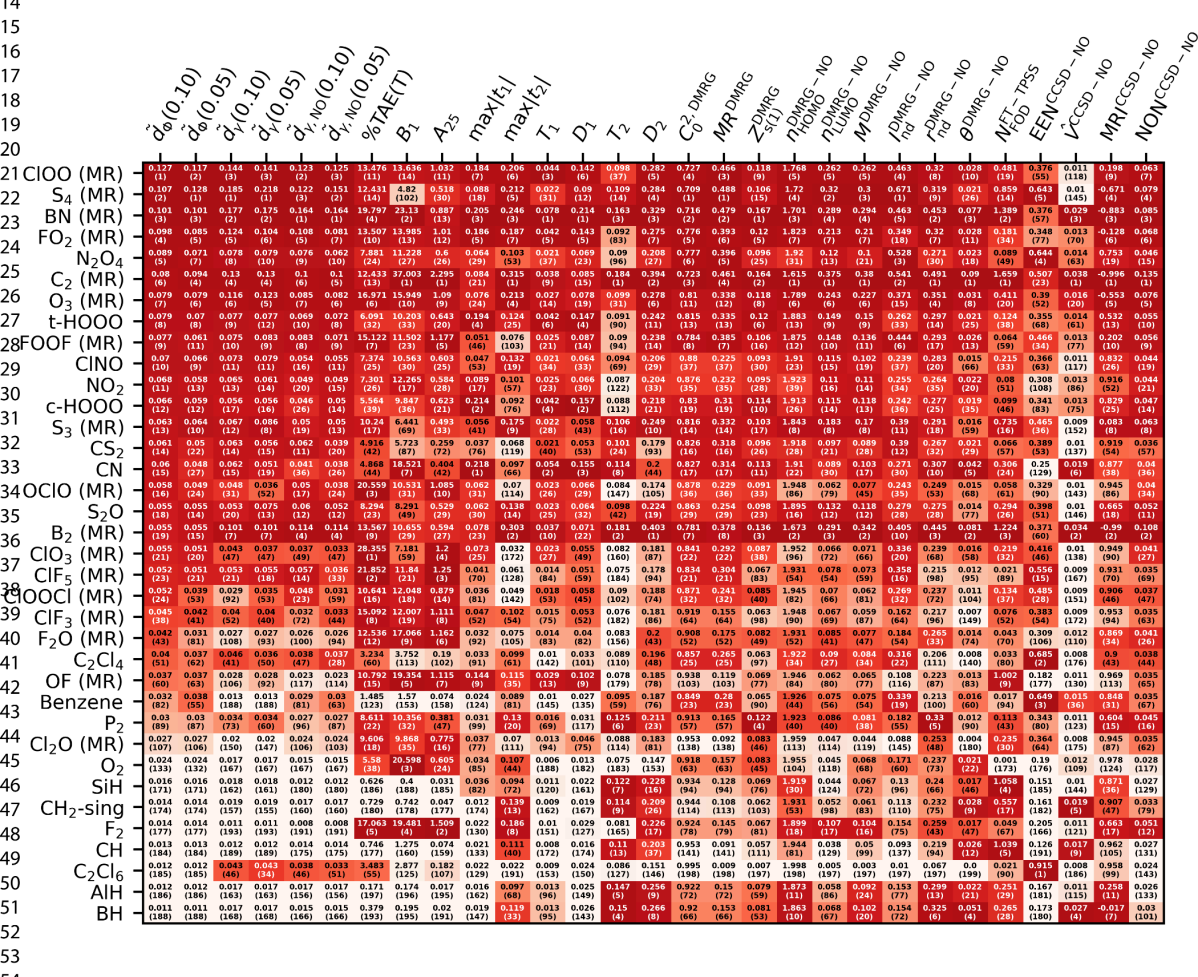
*d̃* and Other Commonly Used MR Diagnostics for a Selected Subset of W4–17 Dataset^a^

a It contains the W4-17-MR subset, marked with “MR”, and additional species which has a *d̃* value in the top 15 or other metric which is in the highest 5 among the species of W4-17. The number in the cells shows the value of the corresponding metric, while in parentheses, the rank of this value among the species is presented. For better visibility, the rankings are also represented by the cell color, where a deeper red color means a higher ranking.

The different *d̃* formulations consistently identify species with high *d̃*. The largest discrepancy is found for B_2_, where *d̃*_Φ_(0.05) gives 0.055, which can be considered a relatively high value among *d̃*_Φ_-s, while *d̃*_γ_(0.05) and *d̃*_γ,NO_(0.05) produce 0.101 and 0.114, respectively. Other notable differences are benzene with a relatively small *d̃*_γ_ (0.013) and C_2_Cl_6_ with a relatively high *d̃*_γ_ (0.043) compared to *d̃*_Φ_ values (0.038 and 0.012) and rankings.

In general, the looser and tighter thresholds for active space produce similar results, but the different formulations differ considerably. *d̃*_Φ_-s are smaller than *d̃*_γ_ due to the fact that *c* coefficients are used up to double excitations. Another thing that was noticed earlier was that NO-transformation also leads to smaller *d̃* values, even though we expect similar results. A more detailed statistical analysis will be discussed later.

Considering the rest of the metrics, there are numerous discrepancies in the studied species. From ClOO to B_2_ (upper half of the table) most diagnostics and *d̃* have high values, but max|*t*_2_|, *T*_2_, EEN, and *V̂* disagree with them notably. Regarding the W4–17 classification, which is based on the %TAE­(T) diagnostics, N_2_O_4_, t-HOOO, c-HOOO, NO_2_, S_2_O, CS_2_, and CN are species with a notable *d̃* (higher than 0.05) but are not part of the MR subset. However, OClO, ClOOCl, ClF_3_, F_2_O, OF, and Cl_2_O all have *d̃* (0.05)-s less than 0.05, although they are part of the W4–17-MR subset. Hydrides are interesting cases because *d̃* and energy-based metrics show low values, but *T*_2_, *D*_2_, *n*_HOMO_, *M*, *I*_nd_, *r*_nd_, θ, FOD, *V̂*, and MRI show significant MR character. For energy-based diagnostics, Cl_2_O, O_2_, and F_2_ seem to be problematic cases.

**Table 3:**
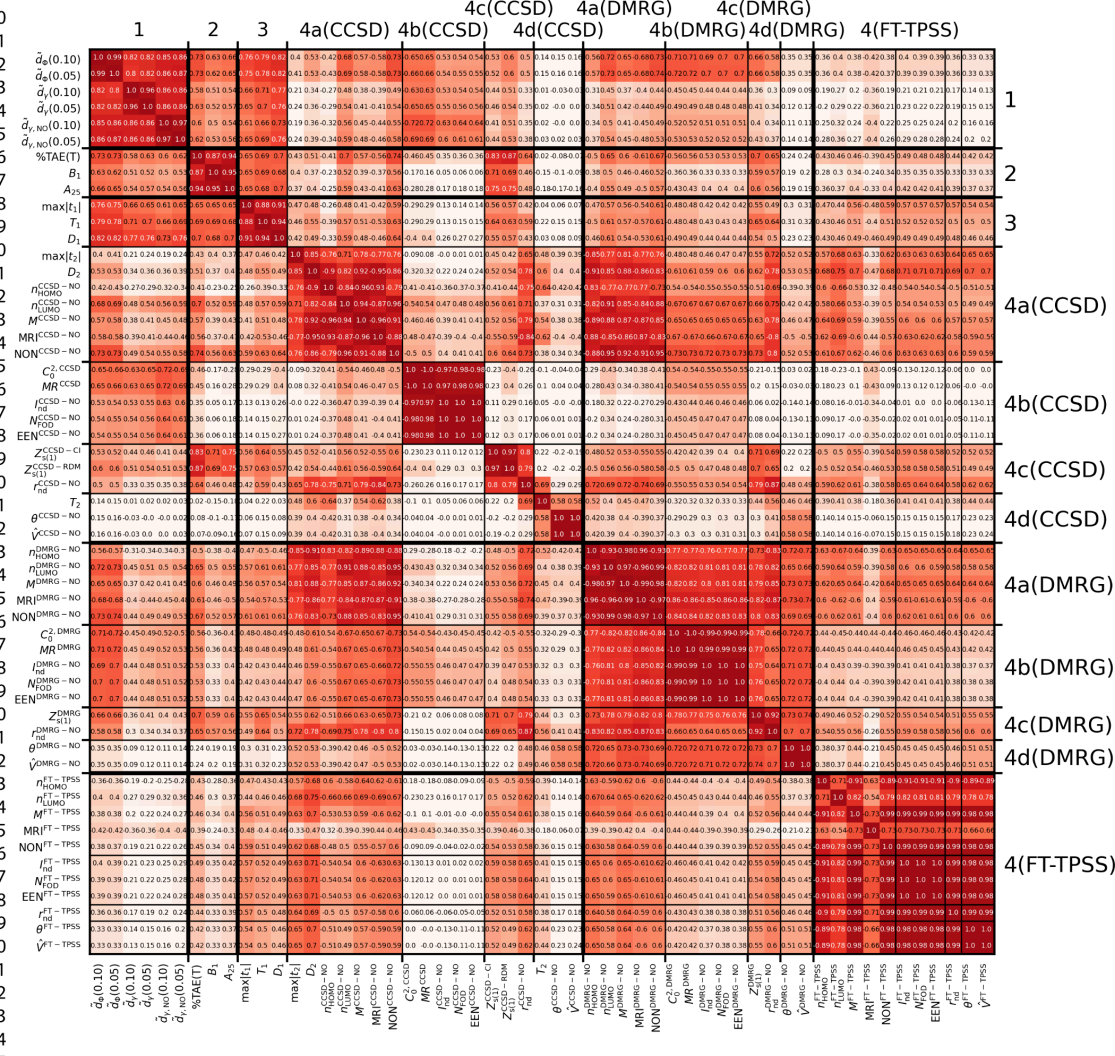
Pairwise Spearman Rank Correlation Matrix of *d̃* and Other MR Diagnostics for the W4–17 Dataset^a^

a The more intense red hue represents a higher correlation. The left and lower labels show the metrics, while the right and upper labels show the corresponding groups.

To better understand the practical relationship between the metrics, we determined the pairwise correlation coefficients (*r*, ρ, and τ). Table 3 shows the Spearman rank correlation matrix, while the other two (*r*, τ) can be found in SI.

First, we look at the correlation between the *d̃* metrics. The correlation between the same metrics with different active space thresholds is close to 1 (*r* = 0.97–0.98, ρ = 0.97–099, τ = 0.87–0.94). However, between the three different types of *d̃* metrics, there is a lower but still strong correlation. *d̃*_Φ_–*d̃*_γ_ have *r* = 0.86–0.91, ρ = 0.80–0.82, τ = 0.63–0.66; *d̃*_Φ_–*d̃*_γ,NO_ have *r* = 0.86–0.90, ρ = 0.85–0.87, τ = 0.68–0.70; and *d̃*_γ_–*d̃*_γ,NO_ have *r* = 0.92–0.96, ρ = 0.86, τ = 0.69–70.

Based on these statistics, the metrics can be categorized into a few groups (see Table 4). The different *d̃* metrics are in their own category (group 1) and do not correlate with any other metrics, which is understandable because they are totally different from the others and try to answer a different question. Energy-based descriptors (%TAE­(T), *B*_1_, *A*_25_) have their own group (group 2). This is not surprising because all of them use total atomization energies. The metrics derived from single amplitudes (max|*t*_1_|, *T*_1_, and *D*_1_) are also separated from the other diagnostics (group 3), which mostly measure orbital relaxation effects. However, for other multireference diagnostics the main distinguishing property is not the formulation but the source of the data, i.e., occupation number and entropy-based diagnostics are grouped by if they derived from CCSD, DMRG, or FT-DFT. Within these groups, the metrics can be further divided.

**Table 4:** Groupings of MR Diagnostics Based on Pearson, Spearman, and Kendall *τ* Correlations of Each Metrics Result on the Species of the W4–17 Dataset

1	2	3	4a	4b	4c	4d
*d̃*_Φ_	%TAE(T)	max|*t*_1_|	max|*t*_2_|	*C*_0_^2^	*Z*_s(1)_	*T*_2_
*d̃*_γ_	*B*_1_	*T*_1_	*D*_2_	*MR*	*r*_nd_	θ
*d̃*_γ,NO_	*A*_25_	*D*_1_	*n*_HOMO_	*I*_nd_		*V̂*
			*n*_LUMO_	*N*_FOD_		
			*M*	EEN		
			NON			
			MRI			

Group 4a contains the simple occupation number-based diagnostics (*n*_HOMO_, *n*_LUMO_, *M*, NON), and surprisingly MRI, which is a complex transformation of them. Also, NON is basically the spin–orbital version of *n*_LUMO_. In group 4b we can see *C*_0_^2^ and MR are closely related, because *C*_0_^2^ is the largest contributor of MR. EEN and *N*_FOD_ are also defined in a way that they are identical, only *N*_FOD_ is half of EEN. *I*_nd_ also has a similar form to *MR* (∑_σ,*i*_ *n*_*i*_^σ^(1 – *n*_*i*_^σ^) = ∑_σ,*i*_ *n*_*i*_^σ^ – *n*_*i*_^σ2^ ∼ ∑_*i*_ |*c*_*i*_|^2^ – |*c*_*i*_|^4^). θ and *V̂* in group 4d also have a similar philosophy by taking the difference of occupation numbers from the ideal and scaling with the number of electrons.

Notice that metrics from double amplitudes can also be sorted in this grouping with other diagnostics derived from CCSD natural orbital occupations. Looking at the off-diagonal elements, we can see that the subgroups of group 4 are fairly correlated for diagnostics derived from DMRG and FT-DFT (except for MRI^FT–TPSS^), while in the case of CCSD, the subgroups are much more separated, especially group 4b. For DMRG, the reason might be that approaching FCI the differences between the various MR definitions are getting smaller. In the case of FT-TPSS data, we can observe that the default settings produce occupation numbers that do not differ from the occupation of the reference state, leading to small separation for SR species and large deviation from other metrics.

Group 1 has some noticeable correlation only with group 3 (*r* = 0.53–0.77, ρ = 0.61–0.82 τ = 0.44–0.63), and *d̃*_Φ_ has noticable correlation with DMRG-based groups 4a and 4b (*r* = 0.67–0.84, ρ = 0.56–0.74 τ = 0.40–0.54). These are not that high; therefore, *d̃* cannot be estimated using other existing MR diagnostics. Another connection can be observed between the members of the CCSD and the DMRG-based subgroup 4a (*r* = 0.68–0.95, ρ = 0.73–0.95, τ = 0.55–0.81).

Earlier comparisons found conclusions similar to those we did. Fogueri et al.^8^ showed that various *A*_λ_ and %TAE formulations correlate well with each other, and *C*_0_^2^ and *M* form another group. Duan and co-workers^6^ studied 15 MR diagnostics and also concluded that the source of the data for the diagnostics is an important factor. They could also distinguish energy-based descriptors from other metrics, such that *T*_1_ and *D*_1_ behave similarly, and *D*_2_ correlates moderately well with other occupation-based diagnostics. Most recently, Martin et al.^40^ studied the correlation between various MR diagnostics and derived TAEx. They determined four clusters: (1) *T*_1_, *D*_1_, max *t*_1_; (2a) *r*_nd_, $\overset{®}{I_{\mathrm{nd}}\;}$ (divided by the number of electrons, not 2, leading to the same expression as *V̂*); (2b) *M*, *D*_2_, max|*t*_2_|; (3) energy-based diagnostics. These groupings are in line with our findings. Xu et al.^93^ showed a connection between $\overset{®}{I_{\mathrm{nd}}\;}$ and *C*_0_, and also between *D*_2_ and maximum of *n*_*i*_^σ^(1 – *n*_*i*_^σ^) values, labeled as *I*_nd_^max^. We already grouped the unscaled *I*_nd_ and *C*_0_^2^ together; however, the size effects are less prominent in the used data set. In a more recent work^94^ they compared several MR diagnostics, and they determined five groups of correlation measures. From these, the dynamic natural orbital occupation (NOO)-based correlation measure $\left(\overset{®}{I_{D}\;}\right)$ is not a good MR diagnostic, while average NOO-based nondynamic correlation measures $(\overset{®}{I_{\mathrm{nd}}\;}$ and $\%I_{\mathrm{nd}}=\overset{®}{I_{\mathrm{nd}}\;}/\left(\overset{®}{I_{\mathrm{nd}}\;}+\overset{®}{I_{d}\;}\right)\times 100)$ could correspond to our 4b group. Maximal nondynamic correlation measures that include *I*_nd_^max^, *n*_*H*_, *n*_*L*_ (maximum and minimum occupancy of occupied and virtual natural spinorbitals), −log­(*I*) (connected to MRI, defined in Table 1), *M*, and *D*_2_ are similar to our group 4a. *D*_1_ and *T*_1_ are in *t*_1_-based correlation measures, as in our group 3, and energy-based correlation measures correspond to our group 2.

Recently, Stanton and his co-workers presented Density Asymmetry Diagnostic (DAD),^95^ which measures the antisymmetry of the 1-RDM of a CC calculaton. In our used code packages (MRCC) such a quantity is not accessible, and only the real symmetrized part of the 1-RDM is returned. Therefore, we did not determine DAD for the studied species but compared our numbers on the W4–11 subset that they used. In this subset, DAD belongs to group 3.

Based on these findings, we can conclude that none of the existing MR diagnostics provides information similar to that of any formulation of *d̃*. However, this is not surprising if we consider that our new metric measures a different property, estimating the distance from the exact solution, while the rest of the MR diagnostics measure the deviation from the ideal single reference solution. Following this line of thought, we are not classifying systems as MR or SR based on *d̃*, but rather evaluate the suitability of the method used, here CCSD. *d̃* increases with the size of the system, and based on the comparison of alkanes and halogenated alkanes (.) found that this scaling is proportional to the number of non-H or heavy atoms (*N*_heavy_). Using this scaling, if *d̃*_γ_ is below $0.03536\times \sqrt{N_{\mathrm{heavy}}}$ , we consider that the used method is highly reliable, while if *d̃* is over $0.07071\times \sqrt{N_{\mathrm{heavy}}}$ , then the used method is not reliable, based on the numerical results of the W4–17 data set. Between the two thresholds, the used method is moderately reliable. The respective thresholds for *d̃*_Φ_ are $0.03\times \sqrt{N_{\mathrm{heavy}}}$ and $0.06\times \sqrt{N_{\mathrm{heavy}}}$ . We note that these thresholds are arbitrary, as most of them do not have strict criteria because we do not have another reference on which we can decide the thresholds. Based on these limits according to *d̃*_γ_(0.05), the calculations of BN, S_4_, C_2_, ClOO, B_2_, and O_3_ are problematic, and FO_2_, S_3_, ClNO, t-HOOO, S_2_O, FOOF, CN, and NO_2_ are moderately difficult for CCSD.

We also note here the differences between the different *d̃*-s. Generally *d̃*_Φ_-s are smaller than *d̃*_γ_, but both have the predictive power to estimate the difference from the FCI. *d̃*_γ,NO_ should lead to a similar value as *d̃*_γ_, but with fewer selected orbitals; however, in some cases, NO-transformation introduces a bias to the original CCSD solution, leading to a more similar DMRG solution and smaller *d̃*_γ,NO_.

We close this section by emphasizing that the *d̃* diagnostic values are clearly attached to the studied calculation. To highlight this important feature, we determined the same *d̃*(0.05) metrics but with CCSD/cc-pVTZ and CCSDT/cc-pVDZ calculation for systems which have *d̃*_γ_(0.05) over 0.075 (Table 5) to see the effect of the basis set and method. As expected, really similar values have been obtained with the larger cc-pVTZ basis set, as with the smaller cc-pVDZ. As for the method, it is clear that eventhough CCSD might be inappropriate in some cases, higher-level methods, like CCSDT, have no problem with the species in question. This is demonstrated by the low (<0.05) values of *d̃*(0.05) for all species and formulations, except S_4_ which have a *d̃*_γ_(0.05) = 0.059 with CCSDT, which is still significantly lower than 0.218 for CCSD. This finding also proves the applicability of high-accuracy thermochemical models like W*n*, HEAT, cccA, etc. for the W4–17 data set.

**Table 5:** *d̃* Values for Species of W4–17 with *d̃*_*γ*_(0.05) over 0.075 for the CCSD/cc-pVDZ Calculations

	CCSD/cc-pVDZ			CCSD/cc-pVTZ			CCSDT/cc-pVDZ		
species	*d̃*_Φ_(0.05)	*d̃*_γ_(0.05)	*d̃*_γ,NO_(0.05)	*d̃*_Φ_(0.05)	*d̃*_γ_(0.05)	*d̃*_γ,NO_(0.05)	*d̃*_Φ_(0.05)	*d̃*_γ_(0.05)	*d̃*_γ,NO_(0.05)
B_2_	0.055	0.101	0.114	0.053	0.121	0.125	0.013	0.023	0.033
BN	0.101	0.175	0.164	0.106	0.195	0.170	0.025	0.028	0.029
C_2_	0.094	0.130	0.100	0.096	0.165	0.149	0.021	0.026	0.026
ClNO	0.066	0.079	0.055	0.065	0.072	0.057	0.028	0.027	0.031
ClOO	0.117	0.141	0.125	0.111	0.140	0.130	0.027	0.026	0.022
FO_2_	0.085	0.104	0.081	0.084	0.103	0.091	0.028	0.028	0.025
FOOF	0.061	0.083	0.071	0.067	0.095	0.066	0.029	0.036	0.029
N_2_O_4_	0.071	0.079	0.062	0.071	0.081^a^	0.062	0.034	0.038	0.047
O_3_	0.079	0.123	0.082	0.082	0.138	0.083	0.026	0.031	0.028
S_2_O	0.055	0.075	0.052	0.062	0.064	0.049	0.026	0.032	0.026
S_3_	0.064	0.086	0.050	0.075	0.105	0.049	0.022	0.036	0.027
S_4_	0.128	0.218	0.151	0.122^b^	0.221^a^	0.158	0.032	0.059	0.059
t-HOOO	0.070	0.077	0.072	0.071	0.076	0.070	0.022	0.028	0.026

a *d̃*_γ_(0.07).

b *d̃*_Φ_(0.07).

### 4.2 Application of the *d̃* Metric to Transition Metal Species

The multireference character among the transition metal species is much more common due to the several low-lying states. In these cases, choosing a reliably accurate, yet numerically affordable, approach can be more problematic. For main group species, the single reference methods usually give correct results; even sub-kJ/mol accuracy is achievable. However, the usage of multireference methods is more complicated, and there is no gold standard such as CCSD­(T) for single reference systems yet.

In such challenging situations, the usage of *d̃* metrics can be even more helpful to decide if a single reference method is applicable to a transition metal species in question. We demonstrate the concept by applying the *d̃* metric for the 3dMLBE data set^67^ and for species studied with s-ccCA recently^68^ labeled as 3d-TM9 and 4d-TM9 for 3d and 4d transition metal species, respectively. For comparison, results based on the *d̃* analysis are presented in Figure 4. The 3d-MLBE20 data set is divided into two subsets where 7 species are considered as single reference and 13 species are considered MR. In case of the 3d-MLBE20-SR subset *d̃*-s are fairly low, mostly below 0.05, only CuCl and ZnS show moderately high values, *d̃*_γ_(0.05) is 0.066 and 0.059, respectively.

**Figure 4:**
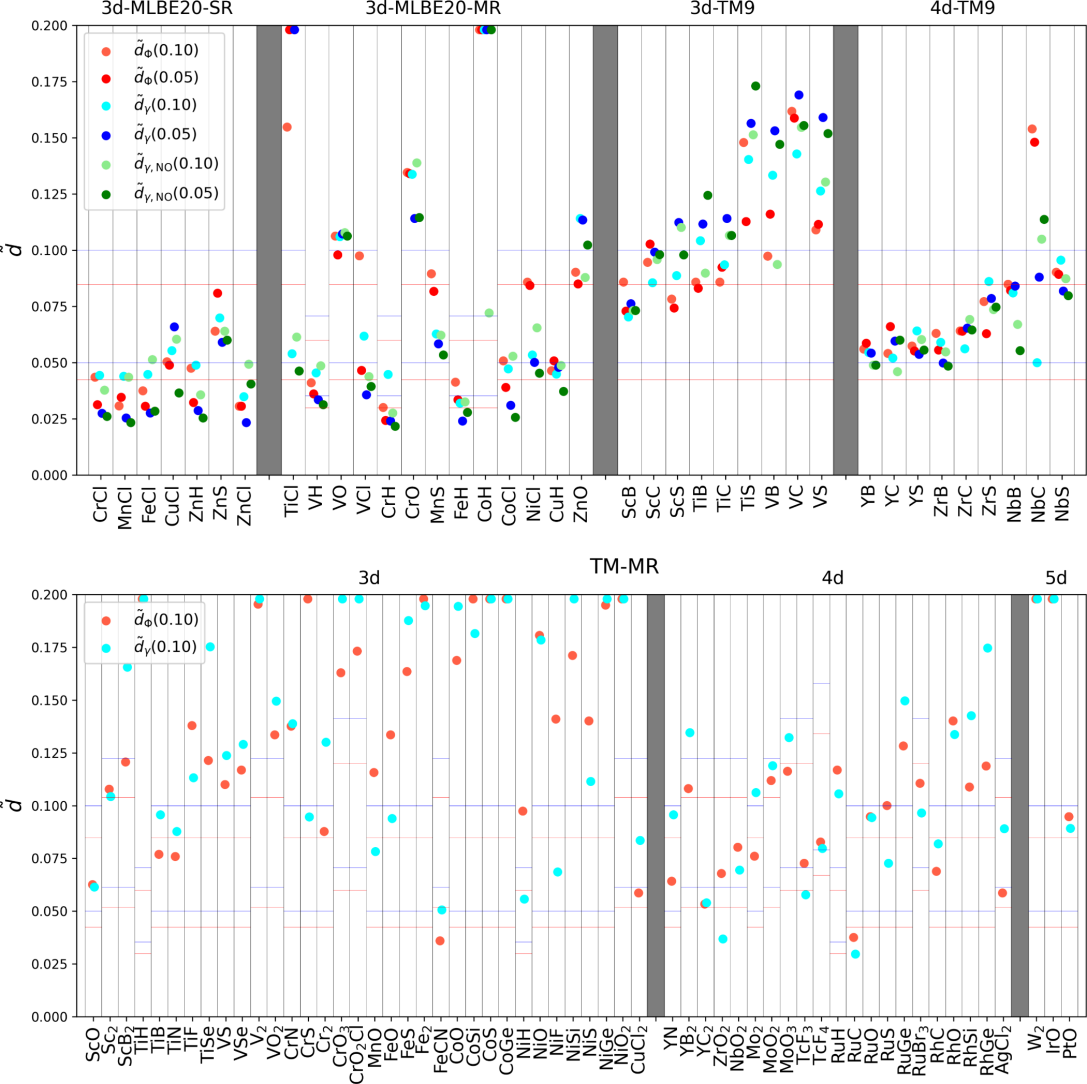
*d̃* values for the TM species studied in this work. Horizontal red and blue lines represent the lower and upper thresholds for the *d̃*_Φ_ and *d̃*_γ_ values, respectively.

From the 3d-MLBE20-MR subset, VH, VCl, CrH, FeH, CoCl, and CuH all have *d̃*(0.05)-s below the corresponding threshold, showing that CCSD is adequate for studying these systems, although it is in the MR subset. TiCl and CoH have exceptionally high *d̃*_γ_(0.05) values (0.427 and 0.305), but in the case of TiCl *d̃*_γ,NO_(0.05) it completely disagrees (0.045). This is the most spectacular case where the NO-transformation causes such a deviation. VO, CrO, and ZnO also have relatively high *d̃*-s, mostly over 0.1.

Looking at 3d-TM9 and 4d-TM9, in ref 68, the multireference character was classified according to the criteria of *T*_1_ > 0.05/0.045, *D*_1_ > 0.15/0.12, and %TAE­(T) > 10%/10% for 3d and 4d species, respectively. Based on this metric, seven 3d species (ScB, ScC, ScS, TiB, VB, VC, and VS) and zero 4d species are considered to have multireference character. However, every *d̃* is greater than 0.05, suggesting that at most only moderately adequate to use CCSD for them. 3d-TM9 species are especially challenging; there are only two species with *d̃*_γ_(0.05) below 0.1, ScB (0.076) and ScC (0.099). 4d-TM9 species are mostly between 0.05 and 0.10, although clasified as a single reference, NbC even shows a *d̃*_Φ_(0.05) of 0.148.

As an additional challenge, we determined *d̃*_Φ_(0.10) and *d̃*_γ_(0.10) values for species labeled as TM-MR, selected from refs 7, 76, and 77 , which are claimed to be MR systems. Based on the size-dependent thresholds determined in the previous section, FeCN, YC_2_, and RuC have low *d̃*(0.1) values, suggesting that CCSD is adequate for them. For ScO, TiB, TiN, CuCl_2_, YN, ZrO_2_, NbO_2_, MoO_3_, TcF_3_, TcF_4_, RuBr_3_, RhC, and AgCl_2_, both *d̃* variants are between their respective thresholds, meaning that one should be careful using CCSD. Some systems have differing *d̃*_Φ_(0.10) and *d̃*_γ_(0.10) values, placing them on different sides of the higher threshold. These species are CrS, Cr_2_, MnO, FeO, NiH, NiF, Mo_2_, MoO_2_, RuO, RuS, and PtO. The rest of the species are problematic for CCSD, especially TiH, V_2_, CrO_3_, CrO_2_Cl, Fe_2_, CoS, CoGe, CoSi, NiSi, NiGe, NiO_2_, W_2_, and IrO for which one of the *d̃*(0.01)-s values is over 0.2.

Note that, in line with the previous findings,^96–98^ our results show that even if a system is regarded as multireference by some criteria, it may be handled with single reference CC to provide reliable data.

Regarding the composition of active spaces, we found high variance. For simpler systems, the occupied *d* and a few virtual *d* orbitals with low principal numbers of a given TM and some occupied and virtual *s* and *p* orbitals from the ligand is enough based on convergence, but the proper description of more correlated systems asks for orbitals with even higher principal numbers (4*d*, 5*d*, etc.) both for the TM and non-TM atoms.

Before concluding this section, we want to highlight another application of the framework presented in this paper. With DMRG the different excited states can be easily determined, which is useful especially in the case of transition metal species to determine the correct ground state which can be problematic to find. Usually within this framework, such problems are highlighted with extremely high *d̃* values, over 1. In such cases, asking for more eigenvalues from the DMRG calculation and looking at the most dominant determinants can help identify the correct ground state. In this work, we found that the ground state of FeH is not ^4^Δ, but ^6^Δ, of CoCl not ^3^Φ, but ^5^Δ, YC is not ^4^Π, but ^4^Σ, and YC_2_ is not ^2^B_2_, but ^2^A_1_. Note that this is only true for cc-pVDZ basis set; it is possible that the order of these closely lying states are changing with increased basis sets. However, such cases require a more careful approach and study of the possible occupations.

## 5 Conclusion

In this work, we present a new strategy to determine the quality of post-HF wave functions. Instead of trying to measure the deviation from the ideal single reference picture, as many multireference diagnostics do, we measure the difference from an approximate FCI solution. Here, we focus on the CCSD to demonstrate the applicability of the workflow. To this end, a subset of orbitals is chosen based on the CCSD solution, and a DMRG calculation is performed on them. Then the wave functions up to double excitations, Φ or 1-body RDM-s, γ, are compared from DMRG and CCSD to give a single metric labeled as *d̃*_Φ_ and *d̃*_γ_.

We also introduced an orbital selection procedure that ensures that the selected subspace correctly represents the entire space. During this procedure, the orbitals are ranked by their relative importance derived from the CCSD solution, and on the selected subspace, another CCSD is performed, and the Φ-s or γ-s are compared to the original CCSD solution. The subspace is increased until the two CCSD solutions are similar enough. This method enables us to tune the accuracy of the *d̃*. The usage of natural orbitals from the CCSD before orbital selection was also tested, assuming it helps to select smaller subspaces.

Three different workflows were tested on the W4–17 data set and compared with other popular multireference diagnostics. The three flavors of *d̃* correlate well with each other (*d̃*_Φ_, *d̃*_γ_, and *d̃*_γ,NO_). *d̃*_Φ_ is usually smaller than *d̃*_γ_; therefore, with the same active space selection threshold, γ-based selections are somewhat larger. However, NO-transformation produces smaller active spaces, but also introduces a bias to the CCSD solution that leads to smaller *d̃*_γ,NO_ than *d̃*_γ_.

None of the metrics were found to correlate well with *d̃*-s. MR diagnostics were also grouped by their performance and found that the source of the data is as important as the formulation of the MR diagnostic itself. The following groups were determined, which are in line with previous findings: group 2: %TAE­(T), *B*_1_, *A*_25_ ; group 3: max|*t*_1_|, *T*_1_, *D*_1_; group 4a: *n*_HOMO_, *n*_LUMO_, *M*, MRI, NON, max|*t*_2_|, *D*_2_; group 4b: *C*_0_^2^, *MR*, *I*_nd_, *N*_FOD_, EEN; group 4c: *Z*_s(1)_, *r*_nd_; group 4d: θ, *V̂ T*_2_.

The proposed approach was also tested on transition metal species. VH, VCl, CrH, FeH, CoCl, and CuH have *d̃*-s below 0.05 with CCSD, showing that it is an adequate method to use on them, despite being in the MR subset. All 3d-TM9 and 4d-TM9 species have *d̃*_γ_(0.05)-s over 0.05 highlighting, which means these are at least moderately challenging. Additionally, 3d species are above 0.1, with the exception of ScB; therefore, CCSD is not recommended to study them. For a selection of MR transition metal species, we found that the calculation of FeCN, YC_2_, and RuC are not problematic, and ScO, TiB, TiN, CuCl_2_, YN, ZrO_2_, NbO_2_, MoO_3_, TcF_3_, TcF_4_, RuBr_3_, RhC, and AgCl_2_ are only moderately difficult for CCSD.

With regard to FeH, CoCl, YC, and YC_2_ molecules, we predicted ground states different from those of the literature findings; nevertheless, these results are inconclusive because of the potential basis set sensitivity of the problems. However, our results demonstrate that the proposed procedure is also capable of finding the correct ground state, which is not always straightforward in transition-metal chemistry.

Based on the results, we find that in the case where *d̃*_γ_ (*d̃*_Φ_) is below $0.03536\times \sqrt{N_{\mathrm{heavy}}}$ $\left(0.03\times \sqrt{N_{\mathrm{heavy}}}\right)$ , the method used is adequate, while for ${\mathrm{d̃}}_{\gamma }>0.07071\times \sqrt{N_{\mathrm{heavy}}}$ $\left({\mathrm{d̃}}_{\Phi }>0.06\times \sqrt{N_{\mathrm{heavy}}}\right)$ the used method is not reliable and other approaches are suggested to study the system in question. In the intermediate region of *d̃*, one should be cautious with the derived results. It must be noted that these limits are not strict, just recommendations based on the results in this work.

The approach presented here can be applied to test the quality of any wave function method where CI coefficients or RDMs can be extracted and a proper active space can be selected. In the matter of high-level reference methods, DMRG can be substituted for any reference-free method that is capable of providing an approximate FCI solution for large enough active spaces. This way the approach has the potential to become a quality assurance tool for wave function based methods.

## Supplementary Material







## Notes

Results for W4–17 and transition metal species, MR diagnostics for W4–17, and Pearson, Spearman, Kendall correlation statistics between the diagnostics (XLSX)
Geometries of the studied species (ZIP)
Validation of the introduced approximations and additional figures and tables (PDF)
