# Supplementary material for: Assessing the Reliability of Truncated Coupled Cluster Wave Function: Estimating the Distance from the Exact Solution
Source: J Chem Theory Comput. 2025 Sep 12;21(18):8737–52. doi: 10.1021/acs.jctc.5c00750 (PMC12461942; doi:10.1021/acs.jctc.5c00750)
Supplement: Supplementary file 3 [file ct5c00750_si_003.pdf]

Supporting Information for "Assessing the Reliability of Truncated Coupled Cluster Wave Function: Estimating the Distance from the Exact Solution"

Ádám Ganyecz,[*,†] Zsolt Benedek,[‡,¶] Klára Petrov,[§] Gergely Barcza,[†] András Olasz,[†,∥] Miklós A. Werner,[†] and Örs Legeza[†,⊥,#]

†*Strongly Correlated Systems "Lendület" Research Group, Wigner Research Centre for Physics, H-1525, Budapest, Hungary*

‡*Department of Physics of Complex Systems, Eötvös Loránd University, Egyetem tér 1-3, H-1053 Budapest, Hungary*

¶*MTA–ELTE Lendület "Momentum" NewQubit Research Group, Pázmány Péter, Sétány 1/A, 1117 Budapest, Hungary*

§*Department of Physical Chemistry and Materials Science, Faculty of Chemical Technology and Biotechnology, Budapest University of Technology and Economics, Műegyetem rkp. 3., H-1111 Budapest, Hungary*

∥*Furukawa Electric Institute of Technology Ltd., Késmárk street 28/A, H-1158 Budapest, Hungary*

⊥ *Institute for Advanced Study, Technical University of Munich, Lichtenbergstrasse 2a, 85748 Garching, Germany*

#*Parmenides Stiftung, Hindenburgstr. 15, 82343, Pöcking, Germany*

E-mail: ganyecz.adam@wigner.hun-ren.hu

Contents of Supporting Information:

- List of all studied species in Table S1.
- Validation of the introduced approximations (Section S1)
- Pearson, Spearman, Kendall correlation statistics (Table S3-S4.)
- Comparison of $\tilde{d}$-s for alkanes and halogenated alkanes (Table S5.)

Table S1: List of all studied species. For TM species the used state is also presented, if the used state differs from what is listed in the source then state in the reference is also shown in parantheses.

**W4-17 (SR)** Ref. 1					
Acetaldehyde	C_2F_6	CH_4	Formic acid	HS	Propane
Acetic acid	C_2H_2	CHF_3	Formic-anhydride	Ketene	Propene
AlCl	C_2H_3F	cis-$C_2F_2Cl_2$	Furan	Methanol	Propyne
$AlCl_3$	C_2H_4	Cl_2	Glyoxal	n-butane	Pyrrole
AlF	C_2H_5F	ClCN	H_2CCN	n-pentane	S_2
AlF_3	C_2H_6	ClCOF	H_2CN	N_2	S_2O
AlH	CCH	ClF	H_2CO	N_2H	SF_6
AlH_3	CCl_2	ClNO	H_2NO	N_2H_4	Si_2H_6
Allene	CCl_2H_2	ClO	H_2O	N_2O	SiF
Allyl	CCl_2O	CN	H_2S	N_2O_4	SiF_4
B_2H_6	CCl_3H	CO	HCCF	NCCN	SiH
Benzene	CCl_4	CO_2	HCl	NH	SiH_3F
Beta-lactim	$CClH_3$	CS	$HClO_4$	NH_2	SiH_4
BF	CF	CS_2	HCN	NH_2Cl	Silole
BF_3	CF_2	Cyclobutadiene	HCNH	NH_2F	SiO
BH	CF_2Cl_2	Cyclobutane	HCNO	NH_2OH	SO
BH_3	CF_4	Cyclobutene	HCO	NH_3	SO_2
BHF_2	CH	Cyclopentadiene	HCOF	NO	SO_3
BN $^3\Pi$	CH_2 $^1A_1$	Cyclopropane	HF	NO_2	SSH
Borole	CH_2 $^3B_1$	Cyclopropene	HNC	O_2	trans-butadiene
cis-HCOH	CH_2C	Cyclopropyl	HNCO	OCS	trans-HCOH
cis-HONO	CH_2CH	Dioxetan-2-one	HNNN	OH	trans-HONO
cis-HOOO	CH_2ClF	Dioxetane	HNO	Oxadiazole	trans-HOOO
cis-N_2H_2	CH_2F_2	Dioxirane	HOCl	Oxetane	trans-N_2H_2
C_2Cl_2	CH_2NH	Dithiotane	HOClO	Oxirane	Tetrahedrane
C_2Cl_4	CH_2NH_2	Ethanol	$HOClO_2$	Oxirene	Thiophene
C_2Cl_6	CH_3	F_2	HOCN	P_2	trans-$C_2F_2Cl_2$
C_2ClH	CH_3F	F_2CO	HOF	P_4	
C_2ClH_3	CH_3NH	FCCF	HONC	PF_3	
C_2ClH_5	CH_3NH_2	FNO	HOO	PF_5	
C_2F_4	CH_3PH_2	Formamide	HOOH	PH_3	

**W4-17 (MR)** Ref. 1					
B_2	Cl_2O	ClO_3	F_2O	O_3	S_3
BN $^1\Sigma$	ClF_3	ClOO	FO_2	OClO	S_4
C_2	ClF_5	ClOOCl	FOOF	OF	

**3d-MLBE-SR**		**3d-MLBE-MR** Ref. 2			
CrCl $^6\Sigma^+$	ZnH $^2\Sigma^+$	TiCl $^4\Phi$	CrH $^6\Sigma^+$	CoH $^3\Phi$	ZnO $^1\Sigma$
MnCl $^7\Sigma^+$	ZnS $^1\Sigma$	VH $^5\Delta$	CrO $^5\Pi$	CoCl $^5\Delta$ ($^3\Phi$)	
FeCl $^6\Delta$	ZnCl $^2\Sigma$	VO $^4\Sigma^-$	MnS $^6\Sigma^+$	NiCl $^2\Pi$	
CuCl $^1\Sigma$		VCl $^5\Delta$	FeH $^6\Delta$ ($^4\Delta$)	CuH $^1\Sigma^+$	

**3d-TM-9**			**4d-TM-9** (all SR); Ref. 3		
ScB $^5\Sigma^-$ (MR)	TiB $^6\Delta$ (MR)	VB $^7\Sigma^+$ (MR)	YB $^5\Sigma^-$	ZrB $^6\Delta$	NbB $^5\Phi$
ScC $^4\Pi$ (MR)	TiC $^3\Sigma^+$ (SR)	VC $^2\Delta$ (MR)	YC $^4\Sigma$ ($^4\Phi$)	ZrC $^3\Sigma^+$	NbC $^2\Delta$
ScS $^2\Sigma^+$ (MR)	TiS $^3\Delta$ (SR)	VS $^4\Sigma^-$ (MR)	YS $^2\Sigma^+$	ZrS $^3\Sigma^+$	NbS $^4\Sigma^-$

**TM-MR**					
3d species from Ref 4					
ScB_2 $^2A_1$	VSe $^4\Sigma^-$	CrO_3 $^1A_1$	FeS $^5\Delta$	NiH $^2\Delta$	NiGe $^1\Sigma^+$
TiB $^6\Sigma^+$	VO_2 $^2A_1$	CrO_2Cl $^2A'$	CoO $^4\Delta$	NiO $^3\Sigma^-$	
TiSe $^3\Delta$	CrN $^4\Sigma^-$	MnO $^6\Sigma^+$	CoSi $^2\Sigma^-$	NiS $^3\Sigma^-$	
VS $^4\Sigma^-$	CrS $^5\Pi$	FeO $^5\Delta$	CoGe $^2\Sigma^-$	NiSi $^1\Sigma^+$	
4d species from Ref 5					
YN $^1\Sigma$	ZrO_2 $^1A_1$	TcF_3 $^5A_1$	RuO $^5\Delta$	$RuBr_3$ $^6A_1$	RhSi $^2\Sigma$
YB_2 $^2A_1$	NbO_2 $^2A_1$	TcF_4 $^4A_2$	RuS $^5\Delta$	RhC $^2\Sigma$	RhGe $^2\Sigma$
YC_2 $^2A_1$ ($^2B_2$)	MoO_2 $^3B_1$	RuH $^4\Sigma$	RuGe $^3\Delta$	RhO $^4\Sigma$	
species from Ref 6					
3d				4d	5d
ScO $^2\Sigma^+$	TiF $^4\Phi$	FeCN $^6\Delta$	$CuCl_2$ $^2\Pi$	Mo_2 $^1\Sigma^+$	W_2 $^1\Sigma^+$
Sc_2 $^5\Sigma$	V_2 $^3\Sigma$	CoS $^4\Delta$		MoO_3 $^1A_1$	IrO $^2\Delta$
TiH $^4\Phi$	Cr_2 $^1\Sigma$	NiF $^2\Pi$		RuC $^1\Sigma^+$	PtO $^3\Sigma^-$
TiN $^2\Sigma^+$	Fe_2 $^9\Sigma^-$	NiO_2 $^3B_1$		$AgCl_2$ $^2\Pi$	

S1 Validation of the introduced approximations

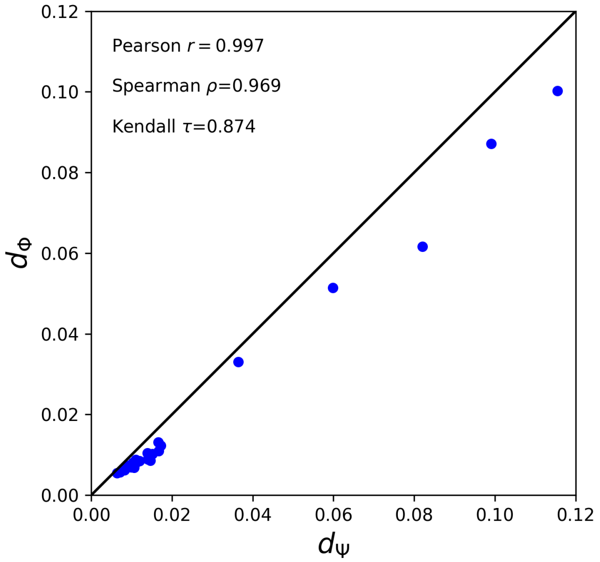

Figure S1: Comparison of d_Ψ values when all coefficients considered and only up to double excitations (d_Φ). Note, that BN, BN $^3\Pi$ and C_2 are only compared up to 7-tuple excitation, NH_3 and PH_3 up to 6-tuple excitations, respectively.

The first approximation used in this work is the truncation of the Ψ wavefunction up to double excitations (Φ), instead of using all the c coefficients due to their large number. To justify the truncation we determined d_Ψ and d_Φ values for species from W4-17 dataset, where FCI reference calculations were possible to perform (AlH, AlH_3, B_2, BH, BH_3, BN, BN $^3\Pi$, C_2, CH, CH_2-singlet, CH_2-triplet, CH_3, H_2O, H_2S, HCl, HF, HS, NH, NH_2, NH_3, OH, PH_3, SiH). In Fig. S1, d_Φ is presented as a function of d_Ψ. It is clear that the truncation leads to lower d values, but d_Φ does not lose its descriptive power ($r = 0.997$, $\rho = 0.969$, $\tau = 0.874$).

The next approximation is the usage of DMRG instead of FCI as an FCI solver. To find the appropriate truncation error for DMRG, which handles its accuracy, we studied the effect of truncation error on d_γ and d_Φ values and presented the error statistics compared to the FCI reference values in Table S2. From this small sample, we can see that d-s can even reach 0.2, but most values are below 0.1. In this case, we would like errors that are at least an order of magnitude smaller, around 0.01. DMRG with 10^{-7} truncation error

Table S2: Error statistics of d_Φ and d_γ with different DMRG truncation errors using FCI results as reference.

truncation error	d_Φ MAX	d_Φ MSD	d_Φ MAD	d_Φ RMSD	d_γ MAX	d_γ MSD	d_γ MAD	d_γ RMSD
10^{-3}	0.2104	0.1237	0.1237	0.1284	0.0999	0.0316	0.0450	0.0488
10^{-4}	0.0607	0.0264	0.0278	0.0324	0.0771	-0.0025	0.0124	0.0202
10^{-5}	0.0057	0.0008	0.0023	0.0029	0.0075	-0.0028	0.0028	0.0032
10^{-6}	0.0073	-0.0001	0.0007	0.0016	0.0014	-0.0005	0.0005	0.0006
10^{-7}	0.0002	-0.0001	0.0001	0.0001	0.0002	-0.0001	0.0001	0.0001

provides basically the same d values as the reference FCI. 10^{-6} and 10^{-5} truncation error gives acceptable error statistics with RMSDs of 0.0016 and 0.0029 for d_Φ, and 0.0006 and 0.0032 for d_γ, respectively. The maximum errors are also smaller than 0.01. However, 10^{-3} and 10^{-4} truncation errors have RMSDs greater than 0.02, which is not acceptable. Considering these, at least a truncation error of 10^{-5} is needed for reasonable results.

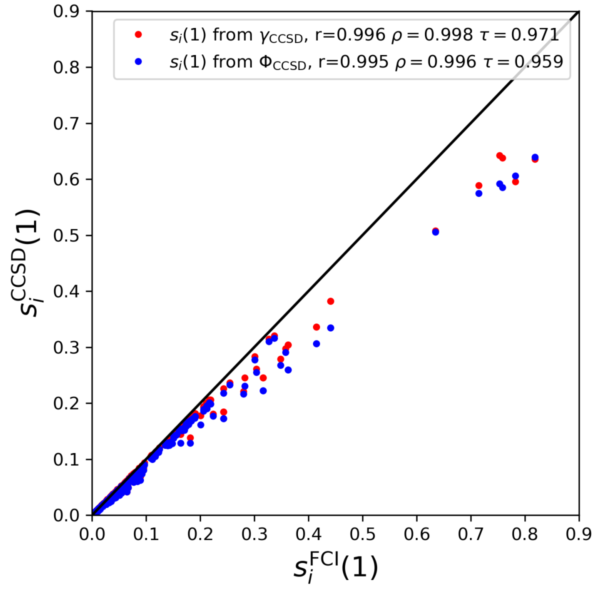

Figure S2: Entropies from CCSD calculations determined from γ_{CCSD} and Φ_{CCSD} compared to entropies from FCI

The third approximation is the usage of a subspace instead of the whole orbital set. The orbital selection procedure is based on $s_i(1)$ values, therefore first we checked how CCSD and FCI entropies relate to each other (Fig. S2). CCSD entropies were determined from both RDM-s and Φ, because this is the data available from the calculation for which d has

to be determined. Generally, entropies from RDM-s of CCSD are smaller, and from Φ-s are even smaller, but they are correlated, ($r > 0.99$, $\rho > 0.99$, $\tau > 0.95$).

In the following, we turn the focus to the analysis of d with respect to the active space size for the three proposed workflows exemplified on the four species with considerable d values (B_2, BN, BN $^3\Pi$, C_2). Due to the active space, we distinguish between d^{a} and d^{b}. In Fig. S3 it can be seen that in general the quality of the active spaces (d^{AS}) is better with more orbitals. Also, using natural orbitals increases the quality even better at first glance. B_2 and BN with canonical orbitals have a bump at 9 and 14 orbitals, respectively. The corresponding d^{a} and d^{b}-s with smaller active spaces differ considerably from the reference values. This local maximum might indicate that not all important orbitals are included. After the local maximum in d^{AS}, the d^{a} and d^{b} values with canonical orbitals are converging to the reference. Although NO transformation produces active spaces with lower d^{AS} and the corresponding d^{a} and d^{b} values have smaller difference between them, but the reference is outside of their interval, especially for C_2. This raises a concern that the NO-transformation might introduce a bias to the CCSD solution leading to smaller d values.

In some cases d^{a} have faster convergence (B_2, C_2), in other cases d^{b} is better (BN$^3\Pi$). However, one can see that the reference value is mostly within the $[d^{\mathrm{b}}; d^{\mathrm{a}}]$ interval, regardless of the active space size. Based on this observation, we introduce their average noted as $\tilde{d}$:

$$\tilde{d}_\Phi = \frac{d^{\mathrm{a}}_\Phi + d^{\mathrm{b}}_\Phi}{2} \tag{1a}$$

$$\tilde{d}_\gamma = \frac{d^{\mathrm{a}}_\gamma + d^{\mathrm{b}}_\gamma}{2} \tag{1b}$$

$\tilde{d}$ have better convergence in most cases and will be used from now, to estimate the d values in question.

However, these systems are too small, therefore we tested the convergence on a larger species with considerable $\tilde{d}$, namely N_2O_4 (Fig. S4).

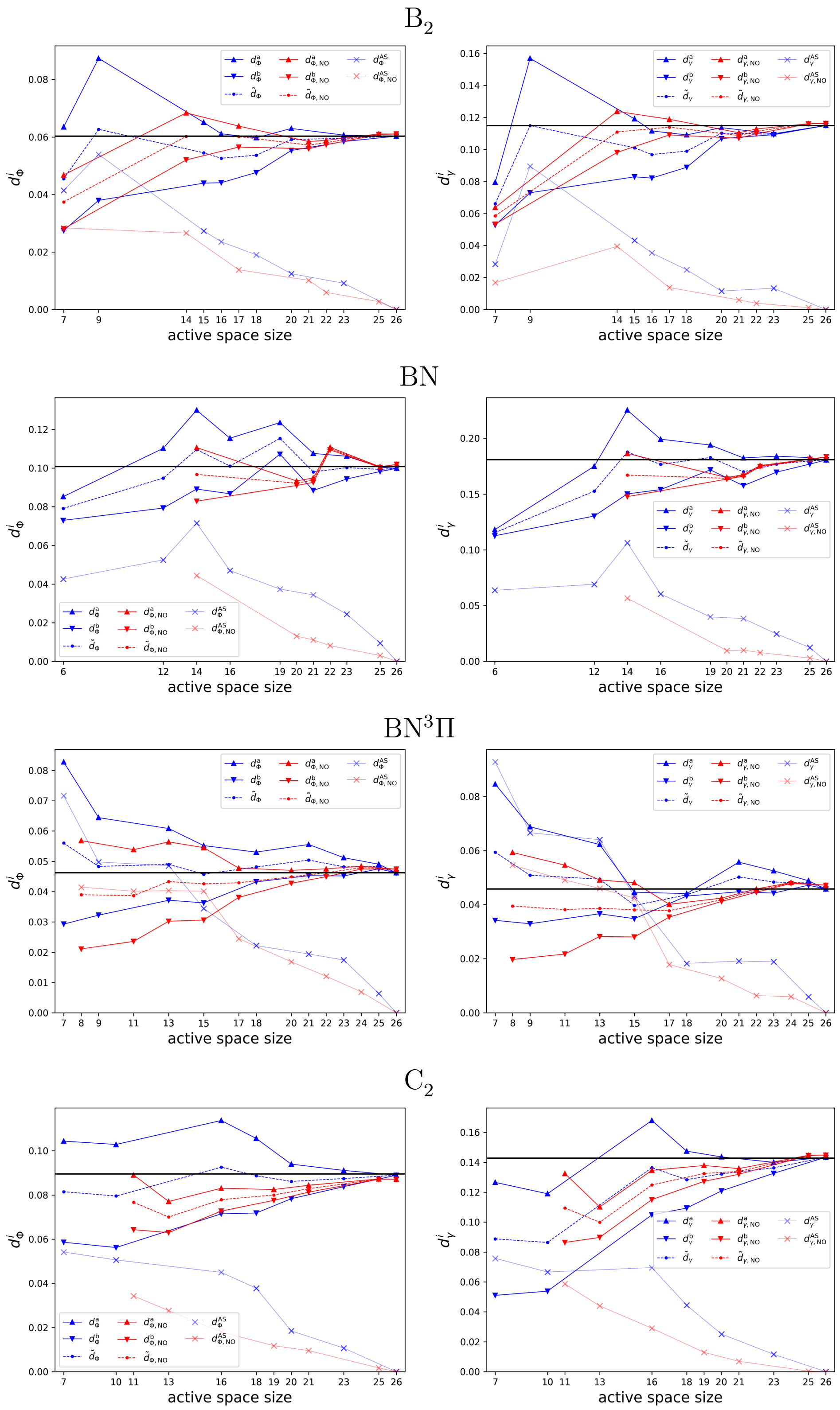

Figure S3: The dependence of d^{a}, d^{b}, and $\tilde{d}$ on active space size for B_2, BN, BN $^3\Pi$, and C_2. Left figures are d_Φ, right figures are for d_γ values, however d-s are determined for all active space selection procedure considered in this work. Upward triangles are for d^{a}, downward triangles for d^{b}, dots are for their average, $\tilde{d}$, and crosses are for d^{AS} values. Blue color represents canonical orbital selection, while red ones used natural orbitals. The black line shows the true d_Φ or d_γ value when all orbital is considered.

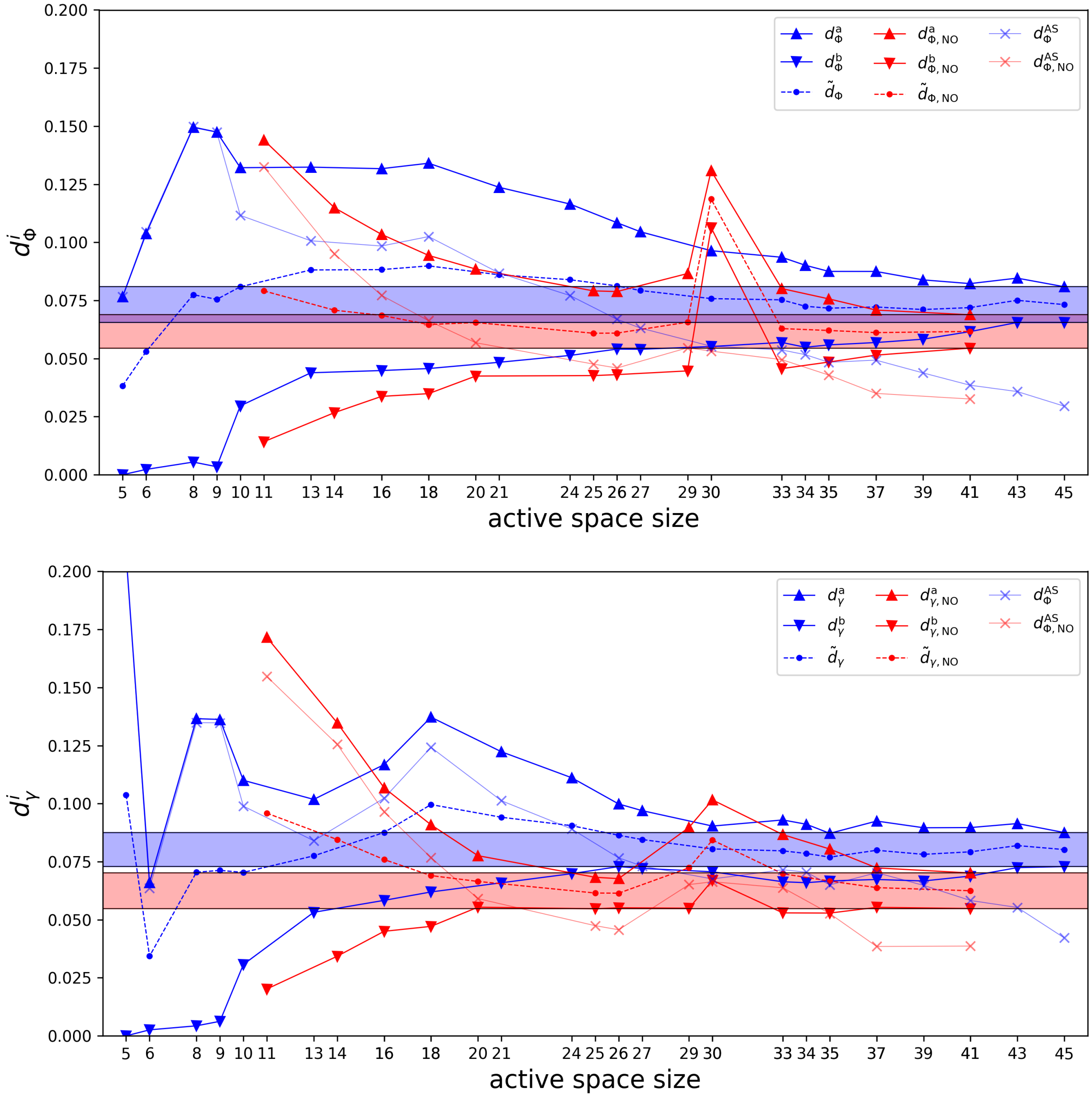

Figure S4: The dependence of d^{a}, d^{b}, and $\tilde{d}$ on active space size for N_2O_4. Upper figure is d_{Φ}, lower figure is for d_{γ} values, however d-s are determined for all active space selection procedure considered in this work. Upward triangles are for d^{a}, downward triangles for d^{b}, dots are for their average, $\tilde{d}$, and crosses are for d^{AS} values. Blue color represents canonical orbital selection, while red ones used natural orbitals The colored bands show the corresponding d with the largest active space as a reference.

Here we can see, that after the local maximum in d^{AS} at 18 orbitals, the canonical d^{a} and d^{b} values are converging to the values from the largest active space with canonical

orbitals. The usage of $\tilde{d}$ is justified here, because its convergence is better than d^{a} and d^{b} for both Φ and γ. In case of natural orbitals, we can see a peak at 30 orbitals for every d-s. There is no obvious explanation for this. Possibly, the added orbital produces an unbalanced orbital set, which leads to a worse wavefunction composition, and has to be accompanied by other orbitals. This shows that there is room for improvement in this simple orbital selection procedure, for example, using mutual information. Besides that, the natural orbital transformation produces better active spaces again ($d^{\mathrm{AS}}_{\gamma,\mathrm{NO}} < d^{\mathrm{AS}}_{\gamma}$), and faster convergence, but $d^{\mathrm{a}}, d^{\mathrm{b}}$ and $\tilde{d}$ are converging to a different value as in the canonical case. This shows again the possible bias of using NO-s from a calculation with questionable reliability that leads to smaller d-s.

Obviously, we cannot perform such analysis in every case, and an appropriate threshold has to be determined for the determination of $\tilde{d}$. To this end, we will use two settings, a looser threshold of 0.1 and a tighter 0.05 for both d^{AS}_{Φ} and d^{AS}_{γ}. The corresponding results will be noted as $\tilde{d}_{\Phi}(0.1)$, $\tilde{d}_{\Phi}(0.05)$, $\tilde{d}_{\gamma}(0.1)$, $\tilde{d}_{\gamma}(0.05)$, where the number in the paranthesis is the used d^{AS} threshold. An additional restriction is that the active space selection error should be lower than with the previous active space candidate, to avoid a selection of active space before a local maximum.

Table S3: Pairwise Pearson correlation matrix of $\tilde{d}$ and other MR diagnostics for the W4-17 dataset. The more intense red hue represents higher correlation. The left and lower labels show the metrics, while the right and upper labels show the corresponding groups.

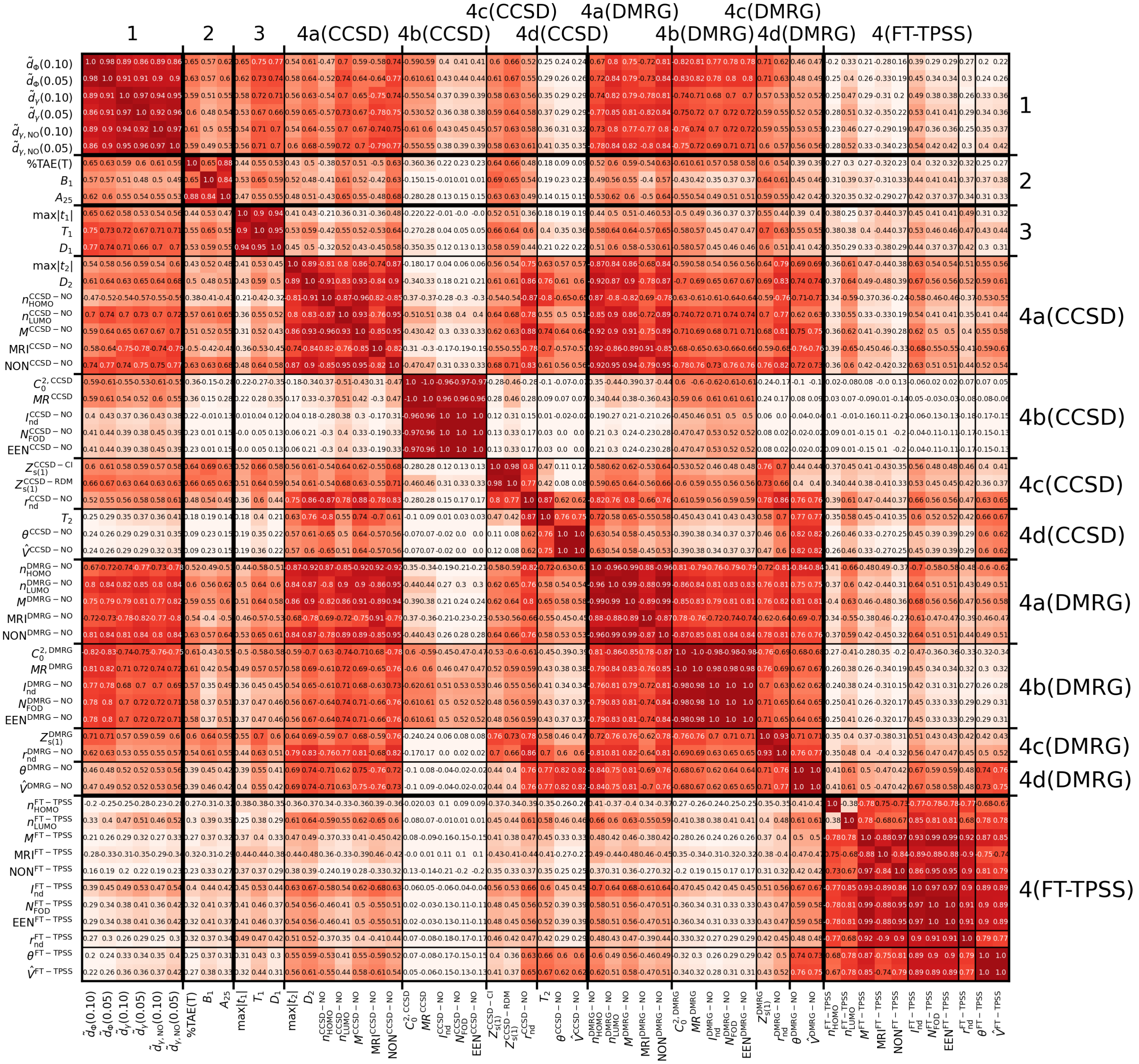

Table S4: Pairwise Kendall rank correlation matrix of $\tilde{d}$ and other MR diagnostics for the W4-17 dataset. The more intense red hue represents higher correlation. The left and lower labels show the metrics, while the right and upper labels show the corresponding groups.

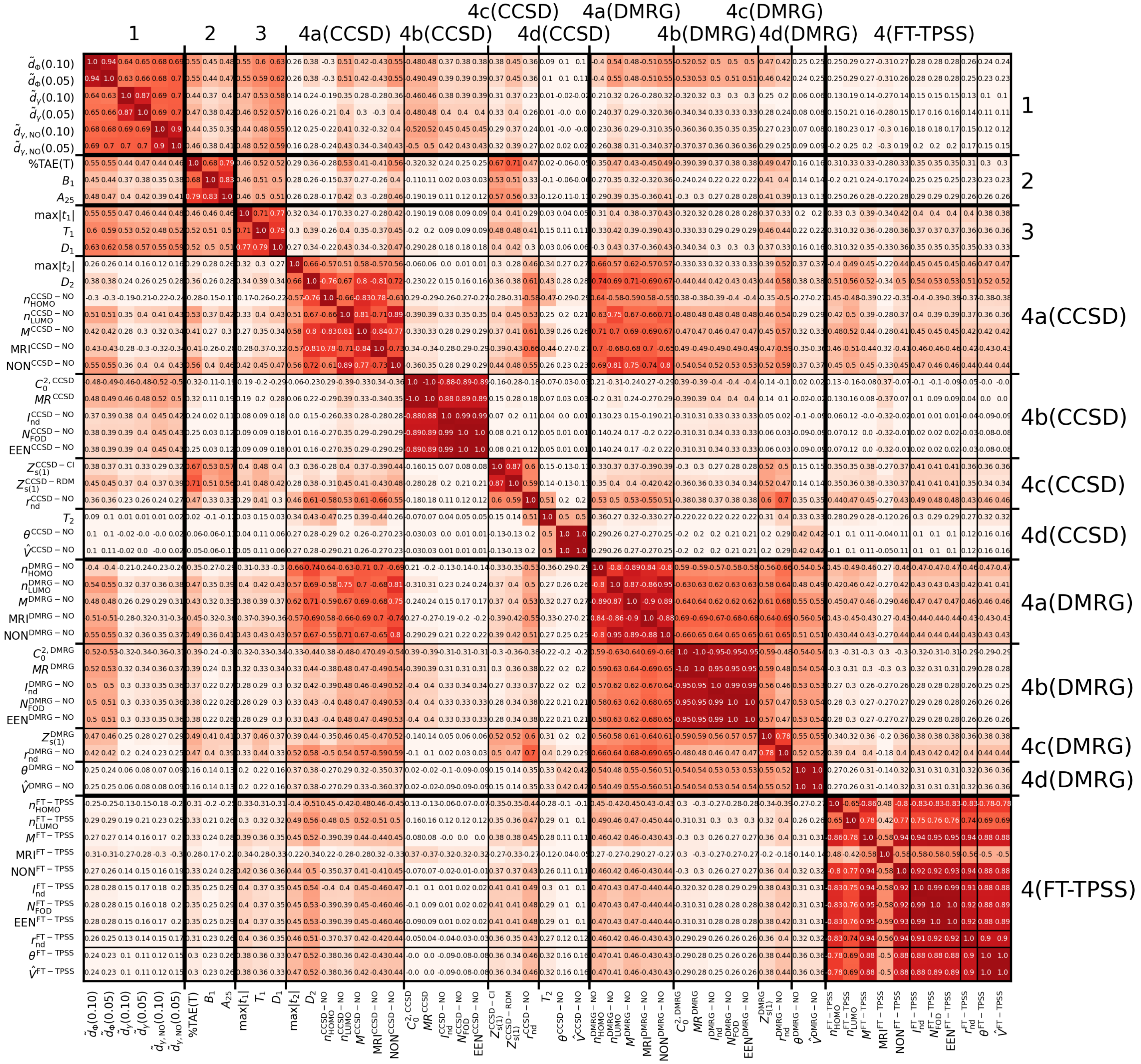

Table S5: Comparison of $\tilde{d}-s$ and their scaled version of alkanes and halogenated alkanes

species	$\tilde{d}_\Phi(0.05)$	$\tilde{d}_\gamma(0.05)$	$\frac{\tilde{d}_\Phi(0.05)}{\sqrt{N_{\text{heavy}}}}$	$\frac{\tilde{d}_\gamma(0.05)}{\sqrt{N_{\text{heavy}}}}$
CH_4	0.013	0.013	0.013	0.013
CF_4	0.027	0.028	0.012	0.013
CCl_4	0.025	0.031	0.011	0.014
CHF_3	0.025	0.029	0.013	0.014
CH_3F	0.017	0.021	0.012	0.015
CH_2F_2	0.019	0.024	0.011	0.014
CH_2ClF	0.021	0.026	0.012	0.015
CF_2Cl_2	0.018	0.036	0.008	0.016
CCl_3H	0.020	0.028	0.010	0.014
$CClH_3$	0.013	0.020	0.009	0.014
C_2H_6	0.011	0.017	0.008	0.012
C_2F_6	0.041	0.045	0.014	0.016
C_2Cl_6	0.012	0.043	0.004	0.015
C_2H_5F	0.021	0.023	0.012	0.013
C_2ClH_5	0.023	0.025	0.013	0.014
C_3H_8	0.024	0.021	0.014	0.012
C_4H_{10}	0.023	0.022	0.012	0.011
C_5H_{12}	0.024	0.023	0.011	0.010

References

(1) Karton, A.; Sylvetsky, N.; Martin, J. M. L. W4-17: A diverse and high-confidence dataset of atomization energies for benchmarking high-level electronic structure methods. *J. Comput. Chem.* **2017**, *38*, 2063–2075.

(2) Xu, X.; Zhang, W.; Tang, M.; Truhlar, D. G. Do practical standard coupled cluster calculations agree better than Kohn–Sham calculations with currently available functionals when compared to the best available experimental data for dissociation energies of bonds to 3 d transition metals? *J. Chem. Theory Comput.* **2015**, *11*, 2036–2052.

(3) Welch, B. K.; Almeida, N. M. S.; Wilson, A. K. Super ccCA (s-ccCA): an approach for accurate transition metal thermochemistry. *Mol. Phys.* **2021**, *119*, e1963001.

(4) Jiang, W.; DeYonker, N. J.; Wilson, A. K. Multireference character for 3d transition-metal-containing molecules. *J. Chem. Theory Comput.* **2012**, *8*, 460–468.

(5) Wang, J.; Manivasagam, S.; Wilson, A. K. Multireference character for 4d transition metal-containing molecules. *J. Chem. Theory Comput.* **2015**, *11*, 5865–5872.

(6) Süß, D.; Huber, S. E.; Mauracher, A. On the impact of multi-reference character of small transition metal compounds on their bond dissociation energies. *J. Chem. Phys.* **2020**, *152*.
